# A careful look at lipid nanoparticle characterization: analysis of benchmark formulations for encapsulation of RNA cargo size gradient

**DOI:** 10.1038/s41598-024-52685-1

**Published:** 2024-01-29

**Authors:** Gretchen B. Schober, Sandra Story, Dev P. Arya

**Affiliations:** 1 NUBAD LLC, Greer, 29650 USA; 2 Department of Chemistry, Clemson University, Clemson, 29631 USA

**Keywords:** Nanoscience and technology, Biophysics

## Abstract

With the recent success of lipid nanoparticle (LNP) based SARS-CoV-2 mRNA vaccines, the potential for RNA therapeutics has gained widespread attention. LNPs are promising non-viral delivery vectors to protect and deliver delicate RNA therapeutics, which are ineffective and susceptible to degradation alone. While food and drug administration (FDA) approved formulations have shown significant promise, benchmark lipid formulations still require optimization and improvement. In addition, the translatability of these formulations for several different RNA cargo sizes has not been compared under the same conditions. Herein we analyze “gold standard” lipid formulations for encapsulation efficiency of various non-specific RNA cargo lengths representing antisense oligonucleotides (ASO), small interfering RNA (siRNA), RNA aptamers, and messenger RNA (mRNA), with lengths of 10 bases, 21 base pairs, 96 bases, 996 bases, and 1929 bases, respectively. We evaluate encapsulation efficiency as the percentage of input RNA encapsulated in the final LNP product (EE_input_%), which shows discrepancy with the traditional calculation of encapsulation efficiency (EE%). EE_input_% is shown to be < 50% for all formulations tested, when EE% is consistently > 85%. We also compared formulations for LNP size (Z-average) and polydispersity index (PDI). LNP size does not appear to be strongly influenced by cargo size, which is a counterintuitive finding. Thoughtful characterization of LNPs, in parallel with consideration of in vitro or in vivo behavior, will guide design and optimization for better understanding and improvement of future RNA therapeutics.

## Introduction

Lipid nanoparticles (LNPs) are promising carriers for therapeutic nucleic acid delivery, with several FDA approved formulations on the market and many in clinical trials^1,2^. LNPs were designed to overcome challenges with delivery of naked nucleic acid payloads, including instability due to degradation by biological machinery and inability to cross membrane barriers. LNPs are synthesized via coassembly, often rapid solvent mixing, of five distinct components: ionizable (cationic) lipid, helper phospholipid, polyethylene glycol (PEG)-lipid, sterol, and nucleic acid. The frontrunner for LNP synthetic methods, with respect to synthesis consistency and ability to scale-up manufacture, is high flow rate microfluidic mixing. This methodology involves the rapid combination of aqueous (containing nucleic acid cargo) and organic (containing lipid mixture) phases, which results in the formation of LNPs with low polydispersity and high encapsulation efficiency^3–7^. Aside from optimization of the synthetic approach, tailoring the lipid composition is arguably the most critical component of LNP formulation^8^.

Each lipid component plays a role in LNP cargo encapsulation and delivery efficiency. Cationic or ionizable lipids (under acidic pH) promote electrostatic interaction with the negatively charged backbone of nucleic acids. This electrostatic interaction facilitates encapsulation of nucleic acid cargo within the electron dense LNP core. Permanently cationic lipids can cause unwanted toxicity and immune response issues, resulting in the increasing adoption of ionizable lipids^9^. Ionizable lipids are positively charged during LNP formation (pKa 6–6.5), while mostly neutral at physiological pH. Maintaining neutral pH during circulation helps prevent adsorption of negatively charged biological molecules, thereby preventing rapid clearance by immune cells and increasing circulation time. The ionizable lipid also facilitates nucleic acid cargo release due to electrostatic interactions with the anionic endosomal membrane, which occurs because of the protonated state of the ionizable lipid under acidic pH within the acidic microenvironment of the endosome^10^. The helper phospholipid (e.g. DSPC, DOPE, DOPC, etc.) increases LNP bilayer stability, which helps prevent leaking of nucleic acid cargo. The phospholipid is also crucial for facilitation of membrane fusion for cellular uptake^11–13^. The steroid component (i.e. cholesterol) impacts bilayer membrane fluidity and permeability, and fills gaps between other phospholipids via hydroxyl head group interaction with aqueous phase of phospholipid membrane. As a result, cholesterol is important in arrangement of the lipid bilayer, and imparts structural stability via tighter lipid packing^14^. The PEG lipid conjugate serves primarily to decrease LNP size, shield the LNP from rapid clearance by reticuloendothelial system, stabilize LNPs via steric repulsion, and prevent protein adsorption due to the hydrophilic chains extending from the surface^15,16^.

Ionizable lipid structure is an active field of research, and has received most of the attention in development of new LNP formulations. Branched lipids, such as ALC-0315 (ALC), have been shown to improve cargo delivery, which is purported to be because of stronger protonation of spaced ionizable lipids, increased cross section of lipid tails, and cone-shaped geometry^17^. The reduction in chain packing and cone-shaped geometry in particular are thought to lead to better fusogenicity. Other structural components of interest include the level and location of unsaturation in the lipid tails^11^, linker moiety between lipid tail and head group^18,19^, and lipid tail length. DLin-MC3-DMA (MC3) is an ionizable lipid with two linoleyl tails, and is used as a benchmark in LNP formulations^20^. Unsaturation in lipid tails generally confers higher membrane fluidity, larger cross section, and has been shown to have a significant impact on cargo delivery^18^. Both ALC and MC3 contain biodegradable ester linkages, which promotes more rapid clearance and reduces potential side effects. These ionizable lipids have been used in combination with cholesterol, DMG-PEG_2000_, and either DSPC or DOPE in FDA approved therapeutic formulations. For example Patisiran (brand name ONPATTRO®) was the first siRNA drug to be approved globally, and is formulated with MC3, DSPC, cholesterol, and DMG-PEG_2000_ for treatment of hereditary variant transthyretin amyloidosis (ATTRv)^21^. The mRNA SARS-CoV-2 vaccine developed by Pfizer-BioNTech contains ALC, cholesterol, DSPC, and a proprietary PEG-lipid^22^. The recipe for successful LNP formulations has become more clear, but there are avenues for improvement that can be identified by a careful characterization of existing formulations.

The bulk of research in this field over the last 10–15 years has been focused on siRNA delivery, while mRNA, ASO, and aptamer delivery has only garnered attention in recent years. Lipid delivery vehicles used for smaller nucleic acid cargo (e.g. siRNA), are not necessarily optimized for delivery of larger RNA cargo (mRNA)^23^. Several factors dictate the potential success of an LNP formulation, but the first steps involve characterization of respective nucleic acid encapsulation efficiency and particle size distribution. Optimizing the efficiency of cargo encapsulation will minimize waste of expensive materials (i.e. RNA), and generate a higher concentration drug formulation. The ionizable lipid is considered to be the most important factor for improving encapsulation efficiency, as this is the component responsible for complexing with RNA cargo. However, while most efforts are focused on altering ionizable lipid structure, the structure and proportion of PEG-lipid, steroid, and phospholipid also require optimization^24^. In addition, the impact of cargo size on nanoparticle diameter is unclear.

**Figure 1:**
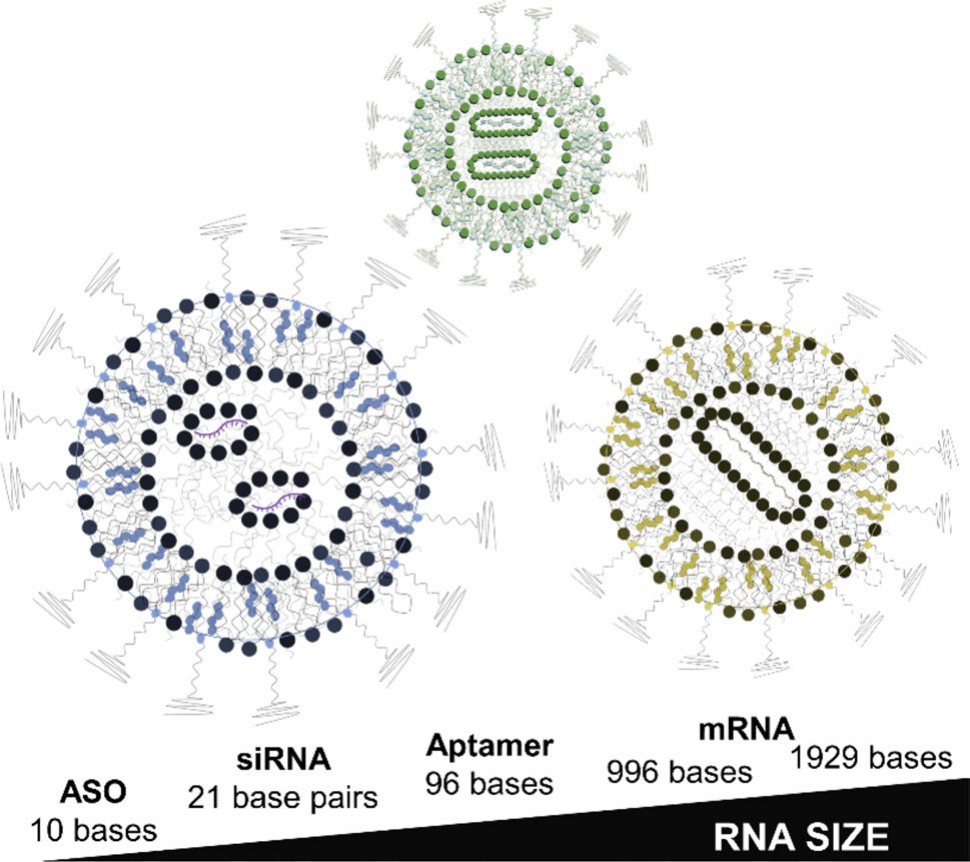
(TOC) Basic illustration of experimental design. Various lipid nanoparticle formulations encapsulate a range of RNA cargo sizes.

To improve and optimize LNP formulation for large-scale production, and achieve desired therapeutic effects, careful examination of benchmark LNP formulations is necessary. To our knowledge there has not been a comparison of the most common LNP formulations for encapsulation of various RNA cargo sizes, under the same reaction conditions. In the present study we compare the encapsulation efficiency, size, and polydispersity index of several benchmark LNP formulations and relevant RNA sizes (Fig. 1). Cholesterol and 1,2-dimyristoyl-rac-glycero-3-methoxypolyethylene glycol-2000 (DMG-PEG_2000_) are kept consistent, while the ionizable lipid and phospholipid components are altered. ALC-0315 and DLin-MC3-DMA are compared as the ionizable lipid component, while DSPC and DOPE are compared as the phospholipid component. The molar ratio of lipid components is kept at 50:38.5:10:1.5 (ionizable lipid, cholesterol, phospholipid, DMG-PEG_2000_), and the ionizable lipid to RNA ratio is kept consistent at 60:1 (w/w) excess.

## Results and discussion

### Encapsulation efficiency characterization

**Figure 2:**
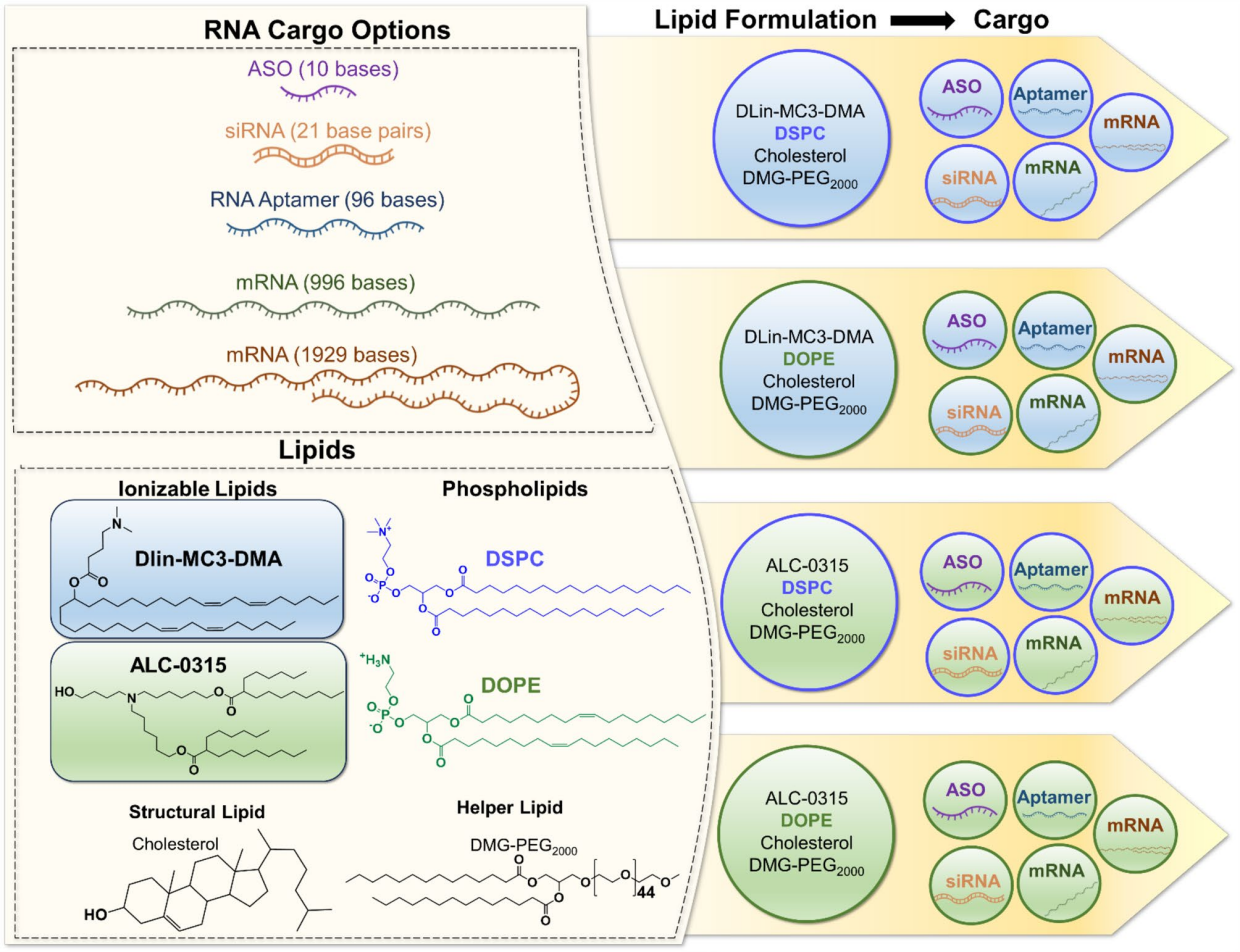
Detailed schematic of experimental design. Lipid nanoparticles (LNPs) are formulated with an ionizable lipid (DLin-MC3-DMA or ALC-0315), phospholipid (DSPC or DOPE), structural lipid (cholesterol) and helper lipid (DMG-PEG2000) at a molar ratio of 50:10:38.5:1.5. Each formulation is tested with 5 different RNA cargo molecules of varying length.

Figure 2 illustrates our experimental design. Four LNP formulations are evaluated for encapsulation efficiency of five different RNA cargo sizes. Recently the benchmark formulation containing an ionizable lipid, phospholipid, cholesterol, and PEG-lipid, has been used for both smaller RNA molecules (e.g. siRNA) and larger RNA molecules (e.g. mRNA), and reported encapsulation efficiency values are greater than 90%. We illustrate a drawback in the traditional calculation of encapsulation efficiency, which can be rectified with a minor change in the calculation.

#### Encapsulation efficiency based on input RNA concentration

While there are a handful of methods to characterize encapsulation efficiency of an LNP formulation for its cargo, modified RiboGreen assay is by far the most common^25–30^. RiboGreen is a dye developed for quantitation of RNA in which fluorescence is produced upon nucleic acid binding^31^. For determination of encapsulation efficiency in LNPs, RiboGreen is first added to the sample with LNPs intact to measure the unencapsulated RNA concentration. A detergent solution (e.g. Triton X-100) is then added to disrupt the nanoparticles, releasing encapsulated RNA, and the total amount of RNA in the sample is calculated from RiboGreen fluorescence. Encapsulated RNA is calculated by subtracting unencapsulated RNA from total RNA and encapsulation efficiency is then taken as the ratio of encapsulated RNA to total RNA in the sample.

Encapsulation efficiency (EE%), as traditionally calculated, describes the percentage of RNA in the sample that is encapsulated by LNPs. However, this ratiometric calculation does not take into consideration the amount of RNA used for the LNP synthesis, and what percentage of that input RNA is encapsulated in the final product. There are several reasons the input RNA concentration should be considered in the evaluation of encapsulation efficiency. Total RNA concentration measured in the sample, post synthesis, is not likely to match the input RNA concentration. RNA, especially shorter strands lacking secondary structure, are delicate and highly susceptible to degradation by RNases^32^. Processing steps after LNP formulation (i.e. buffer exchange, centrifugal concentration steps, etc.) may cause preferential degradation of unencapsulated (unprotected) RNA in the sample due to unintentional RNase contamination. In addition, the synthetic procedure uses microfluidic mixing where rapid flow rates produce shear stress, which may cause mechanical degradation of RNA. This will also decrease the amount of RNA in the sample. These degradation mechanisms will artificially increase encapsulation efficiency by decreasing the unencapsulated RNA concentration.

**Figure 3:**
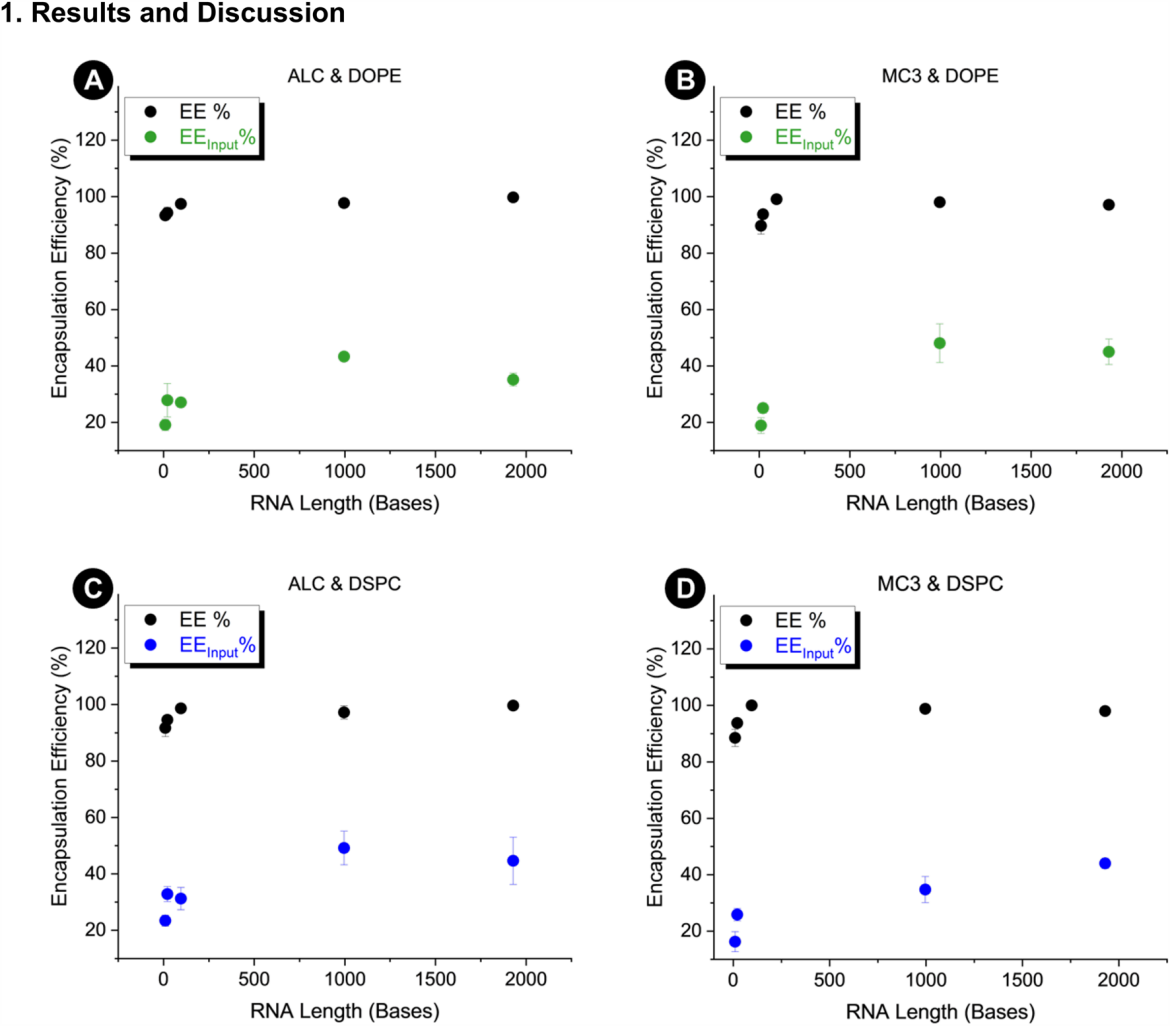
Comparison of standard encapsulation efficiency (EE%) calculation compared to the encapsulation efficiency calculated using input RNA concentration (EE_input_%). EE_input_% reflects the percent of input RNA encapsulated by LNPs in the final product. Standard EE% technically reflects the percent of LNP synthesis output RNA encapsulated by LNPs in final product. Error bars represent standard deviation of six measurements across three samples.

In Fig. 3, encapsulation efficiency is calculated two different ways. First by ratiometrically comparing encapsulated RNA to the total sample RNA concentration (EE%), and second by comparing encapsulated RNA to the input RNA concentration (EE_input_%). EE% is between 88 and 100% for all lipid combinations and cargo types, where EE_input_% is only between 8 and 49%. 96 base RNA encapsulated using the MC3 and DSPC lipid recipe best illustrates the discrepancy, where EE% is 100% but EE_input_%% is only 8%. In all three of these syntheses there is nearly zero unencapsulated RNA detected, meaning that any RNA in the sample is encapsulated by LNPs. According to traditional calculation, this means that encapsulation efficiency approaches 100%. This value is misleading because this synthesis realistically exhibited the lowest encapsulation efficiency with respect to input RNA concentration, yielding only 8% encapsulated. Nearly 92% of input RNA is unaccounted for in the determination of encapsulation efficiency in this example. On average in our study EE_input_% reads 65% lower than EE%, which is significant considering this metric is a keystone in optimization of LNP formulations for therapeutic application. Formulations containing MC3 exhibit 67% discrepancy between EE% and EE_input_%, compared to formulations containing ALC (63%).

A brief follow-up study was performed to investigate whether the discrepancy between EE_input_% and EE% can be reduced. 1929 base mRNA was tested using the same ionizable lipid to RNA ratio (60:1), with several different total lipid concentration values (0.5, 5, and 10 mM). Increasing the total lipid concentration from 0.5 to 10 mM increases EE_input_% from 23 to 82% (Fig. S2). At 0.5 mM, 76% of the input RNA is unaccounted for in the final product, despite traditional calculation suggesting > 75% encapsulation efficiency for all tested samples. These results demonstrate that using a higher total lipid concentration increases the percent of input RNA captured by LNPs, even though the ratio of RNA to ionizable lipid remains constant. Most LNP formulations use a minimum of 10 mM total lipid, and higher concentrations (up to 50 or 60 mM) are common as well^33,34^. However, even though EE% and EE_input_% begin to converge at 10 mM, the existence of discrepancy at any concentration illustrates the need for consideration of input concentration to verify EE% values obtained. By considering input RNA concentration for encapsulation efficiency calculation, in addition to the traditional calculation of EE%, important information about reaction efficiency is elucidated. This additional information can inform formulation and process optimization.

In both EE% and EE_input_%, the general trend is that encapsulation efficiency increases with RNA molecule size. The trend is more apparent in plots of EE_input_% because EE% values approach 100% at ~ 100 bases. For this reason, EE_input_% values will be evaluated to discern differences in encapsulation of RNA cargo by different lipid mixes.

#### Comparison of LNP formulations

Several size RNA molecules were selected to represent the types of cargo investigated for LNP delivery. The size gradient includes an antisense oligonucleotide (ASO) representative, which is a 10-base strand of RNA. Short, single-stranded, antisense oligonucleotides (ASO) are the smallest RNA molecules incorporated into LNPs for the development of new therapeutics, and are designed to reduce protein expression via Watson–Crick base pairing with target mRNA sequences^35^. ASOs are especially vulnerable to degradation due to the lack of secondary structure, so LNPs are an appealing delivery vehicle for this class of RNA therapeutic. Next is a 21-base pair siRNA molecule. LNPs were first introduced as a method for delivery of small interfering, double stranded RNA (siRNA), to silence genes associated with disease pathology^36^. RNA aptamers, which are emerging as a promising alternative to chemotherapeutic agents, are often in the size range of ~ 100 bases^37^. As a result we have included a 96 base RNA to represent potential LNP aptamer cargo. Messenger RNA (mRNA) is the final category, and spans a large size range. These nucleic acids directly code for protein synthesis, and are heavily investigated for protein replacement therapy for genetic disorders^26^, cancer^25^, and immunotherapy^28^. Our representative mRNA molecules are 996 bases, and 1929 bases in length.

**Figure 4:**
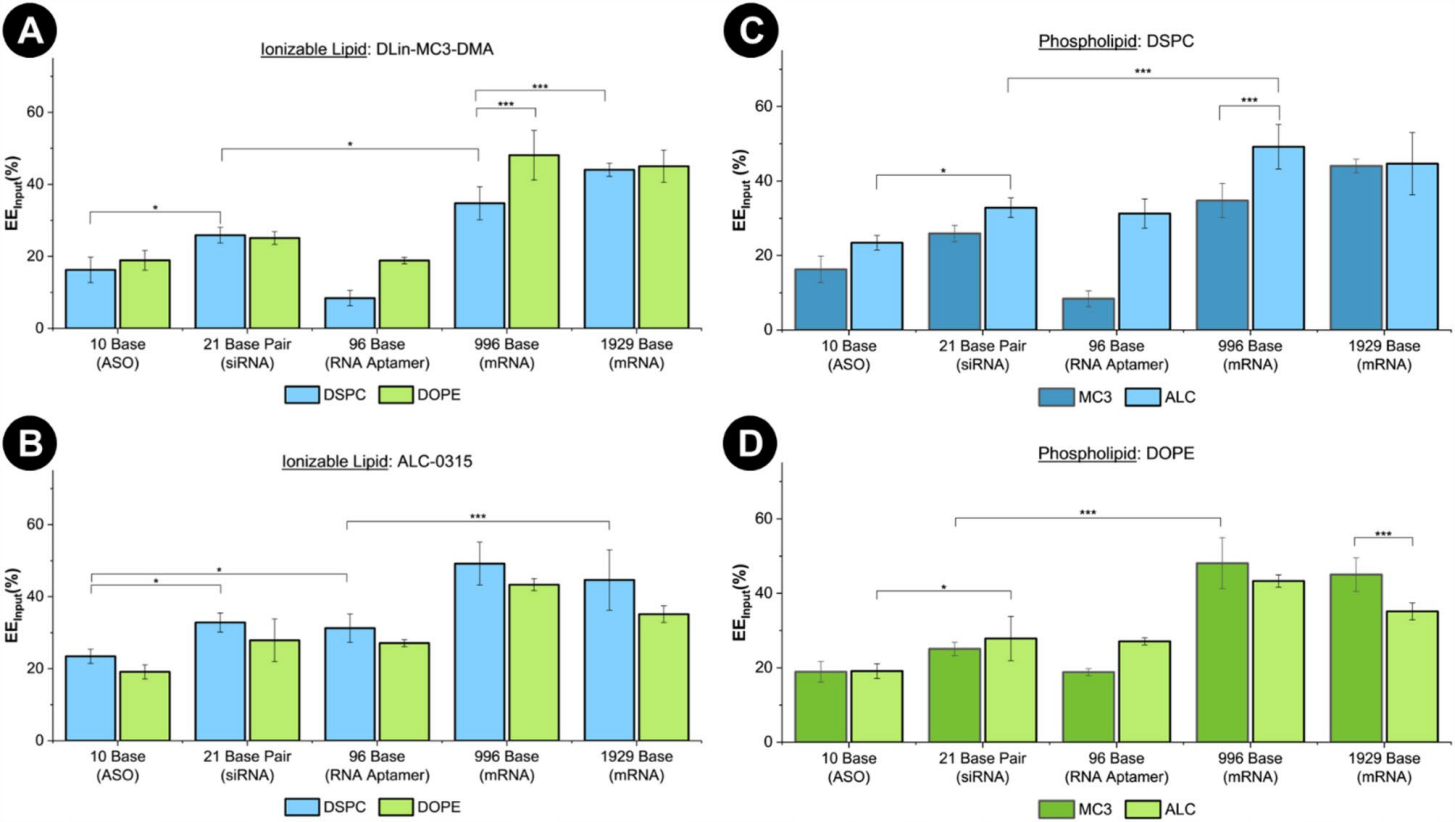
Encapsulation efficiency based on input RNA concentration (EE_input_%) of lipid recipe and RNA combinations. (**A**) Comparison of lipid mixtures containing MC3, with blue bars representing DSPC, and green bars representing DOPE. (**B**) Comparison of lipid mixtures containing ALC, with blue bars representing DSPC, and green bars representing DOPE. (**C**) Comparison of lipid mixtures containing DSPC. (**D**) Comparison of lipid mixtures containing DOPE. Brackets indicate notable significant differences as determined by one-way ANOVA and Tukey test (*P ≤ 0.05, ***P ≤ 0.001).

As mentioned in the previous section, a trend across all lipid formulations tested is that larger mRNA strands are more efficiently encapsulated than the shorter 10–100 base RNA molecules tested. Short (≤ 10 base) RNA sequences are going to exhibit a much smaller net charge, and significant morphological differences compared to a larger RNA aptamer (~ 100 bases) and especially compared to an mRNA molecule (several hundred to thousands of bases). Encapsulation efficiency is largely dictated by the electrostatic interaction between ionizable lipid and the negatively charged RNA backbone. Larger RNA molecules, with a larger net charge, may more efficiently complex with ionizable lipid which would result in a higher encapsulation efficiency. In Fig. 4 the encapsulation efficiency (EE_input_%) is compared for mixtures containing ALC or MC3, and DSPC or DOPE. Comparisons of traditional EE% can be found in Fig. S1. Encapsulation of 10 base ASO ranges from 16 to 23%, 21 base pair siRNA 25–33%, 96 base aptamer 8–31%, 996 base mRNA 35–49%, and 1929 base 35–45%. LNPs formulated with ALC and DSPC show the highest EE_input_% values for all RNA molecules.

### Size analysis and comparison

**Figure 5:**
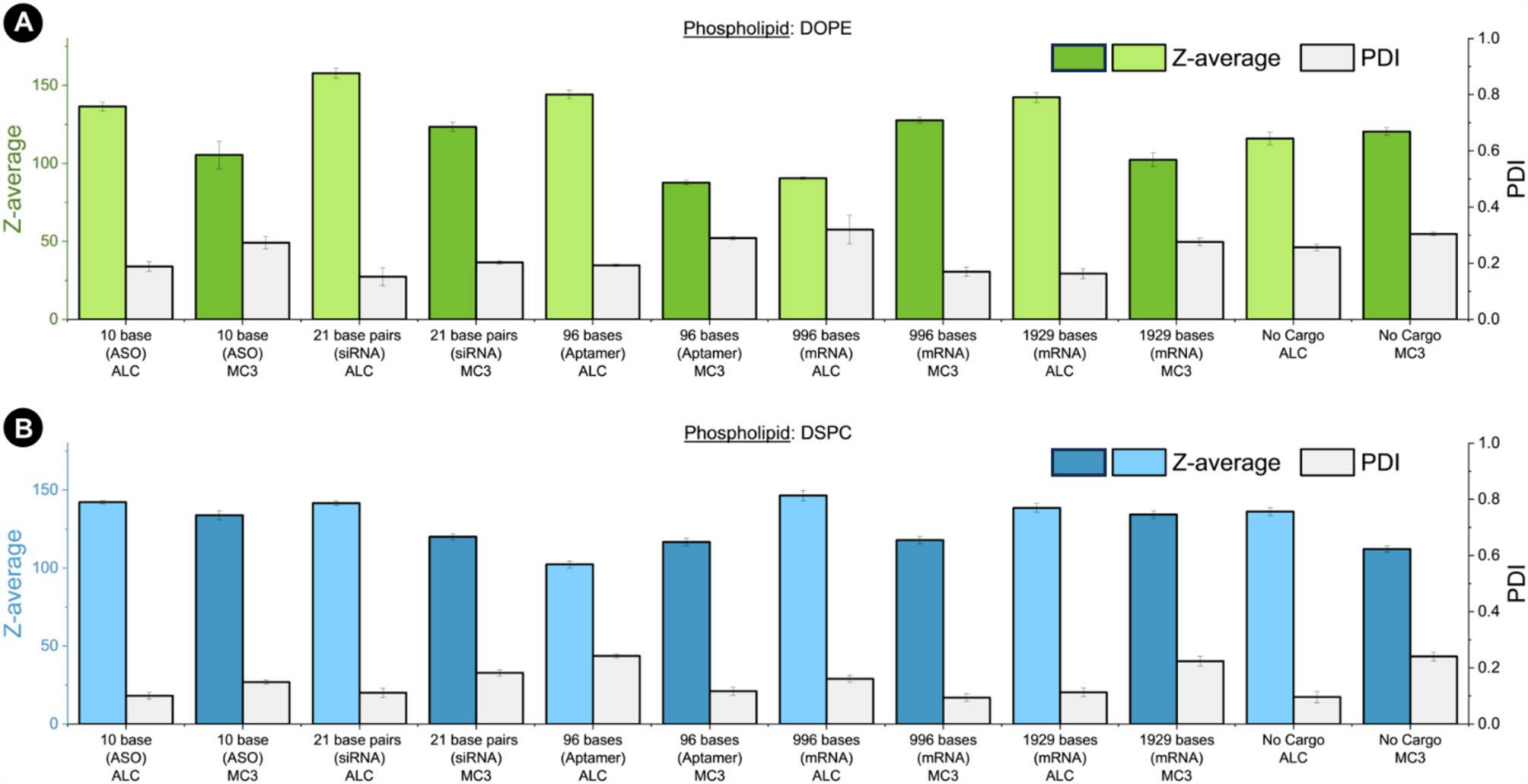
LNP size analysis and polydispersity index values for (**A**) lipid combinations containing DOPE and (**B**) lipid combinations containing DSPC.

Nanoparticle diameter (size) and polydispersity of lipid formulations with various RNA cargo sizes was analyzed (Fig. 5). Polydispersity index (PDI) is a normalized value that indicates nanoparticle size range in a sample, and is a useful indicator of sample quality. In samples with high dispersity, larger particles in the distribution will tend to aggregate and sediment, which leads to diminished effective RNA concentration and inconsistent dosing. Typically, LNP formulations developed for biological application should have a PDI below 0.2, which indicates the colloid is acceptably monodisperse^38^. Monodispersity of nanoparticle drugs is crucial to ensure the consistent behavior of the intended drug, as size influences how particles interact with the body.

DLS size and polydispersity analysis suggests that there is no significant correlation between cargo size and LNP hydrodynamic diameter in the regime of high ionizable lipid excess. This partly agrees with published literature which argues that LNP size is primarily influenced by concentration, flow rate (for particles synthesized using a microfluidic system), and PEG-lipid concentration. However, there has been speculation that differences in cargo size and morphology should affect LNP size^8^. This is because compartmental organization of lipids and cargo within an LNP differs based on cargo size^39,40^. For example, smaller cargo with minimal secondary structure (ASO, siRNA), would likely pack more densely in the LNP core. Whereas larger RNA with complex secondary structure would pack less densely with fewer RNA molecules per LNP. These morphological differences could reasonably exert control over LNP diameter, to some extent. However according to our study, there is no discernable trend between cargo size and LNP size. LNPs without RNA cargo (empty) are generally smaller than particles containing RNA, but there are exceptions. For example, MC3 and DOPE LNPs loaded with 10 base ASO, 96 base aptamer, and 1929 base mRNA are 105 nm, 88 nm, and 102 nm, where the empty particles are 120 nm. However, this further suggests that size is not dictated by cargo size but rather by the mixing parameters and lipid composition.

The use of higher lipid concentration (> 10 mM), and microfluidic controlled rapid mixing, has been shown to push the size of LNPs to the lower limit (below 100 nm and 0.2 PDI). These results support the notion that size is dictated primarily by reaction conditions, and show that cargo size has little effect (using 1 mM lipid mix, and 60 × excess ionizable lipid concentration). For example, LNPs containing 21 base pair siRNA and LNPs containing 1929 base mRNA produce nanoparticles of ~ 140 nm (ALC, DSPC mixture). However, there are some notable differences in size and polydispersity index (PDI) between lipid formulations. LNPs containing DOPE exhibited the highest polydispersity (0.23) across all cargo types, while the formulations containing DSPC exhibit the lowest dispersity at 0.15 (Table 1). ALC-0315 and DLin-MC3-DMA were selected because of their different lipid tail structures. ALC produces LNPs with average size of ~ 133 nm, where MC3 produces LNPs ~ 117 nm. ALC is a larger molecule, with more rigid lipid tails (branched, saturated) that exhibit higher cross section. These particles are expected to be larger than particles made with MC3, which has smaller more fluid lipid tails (unsaturated, no branching). These differences may be exploited to increase or decrease LNP size, when other methods for affecting size are not feasible (lipid concentration or flow rate).

**Table 1:** Average size and PDI values for LNP formulations.

Formulation contains	Z-average (nm)	PDI
DOPE	121	0.23
DSPC	128	0.15
ALC	133	0.17
MC3	117	0.21

## Conclusions

Benchmark LNP formulations have been evaluated for encapsulation efficiency (EE_input_% and EE%) size, and polydispersity of several relevant sizes of RNA molecules. We found that encapsulation efficiency calculated based on input RNA (EE_input_%) is significantly and consistently lower than the corresponding encapsulation efficiency (EE%) calculated according to industry and academic standards. By increasing total lipid concentration, EE_input_% can be increased but does not converge with EE%. There is RNA loss not reported by the traditional calculation of EE%, which is important to consider from a process optimization and manufacturing standpoint. In addition, smaller RNA molecules (e.g. ASO, siRNA) are encapsulated with less efficiency than larger RNA molecules (e.g. mRNA). This is likely due to the difference in morphology and net charge between the two molecules. Concern has been expressed in the literature that formulations designed for siRNA will likely not translate well for larger RNA cargo but the formulation designed and optimized for siRNA (MC3, DSPC, cholesterol, and DMG-PEG_2000_) appears to encapsulate larger RNA more efficiently than smaller RNA. We also find that LNPs synthesized with MC3 are smaller than LNPS synthesized with ALC, which is useful to consider as LNP size dictates in vivo behavior. In general, we find that a more careful characterization of LNP cargo encapsulation and size provides avenues for improvement not usually focused upon. In addition, traditionally calculated EE% values may be misleading and consideration of RNA input concentration provides useful insight for synthesis optimization to reduce RNA loss, and the associated cost.

## Materials and methods

### RNA preparation

1929 base Firefly luciferase mRNA with 5-methoxyuridine modification (FLuc mRNA, L-7202) and 996 base enhanced green fluorescent protein mRNA with 5-methoxyuridine modification (EGFP mRNA, L-7201) are produced by Trilink Biotechnologies (Georgia, USA). 10 base AcpP1 MM2 RNA (5′-AGA CCA UGA G-3′) and 96 base CTLA-4 RNA aptamer (5′ GGG AGA GAG GAA GAG GGA UGG GCC GAC GUG CCG CAA CUU CAA CCC UGC ACA ACC AAU CCG CCC AUA ACC CAG AGG UCG AUA GUA CUG GAU CCC CCC-3′)^41^ custom syntheses performed by Integrated DNA Technologies (Iowa, USA). 21 base pair AccuTarget GAPDH siRNA purchased from Bioneer (California, USA). Concentrated RNA solutions are stored at − 80 °C in DNase/RNase free water, and prepared in sodium acetate buffer (100 mM, pH 4.0) immediately before LNP synthesis. RNA concentration is measured using the NanoDrop One (Thermo Fisher Scientific, Massachusetts, USA).

### Lipid nanoparticle synthesis

d-Lin-MC3-DMA (HY112251) obtained from MedChemExpress LLC (New Jersey, USA). ALC-0315 (890900O), cholesterol (700000P), and DSPC (850365P) are from Avanti Polar Lipids (Alabama, USA). DMG-PEG_2000_ (BP25496) is from BroadPharm (California, USA). DOPE obtained from Tocris Bioscience (Minnesota, USA). Nanoparticles are prepared using a glass, staggered herringbone, microfluidic mixing chip with etched channel depths of 125 µm, and hydrophilic coating (Part number 3200401, Dolomite Microfluidics, England, UK). The chip has three inlets that immediately converge for mixing, with aqueous phase (containing RNA) entering on each side, and the organic phase (containing lipid mixture) entering in the center channel. Lipid mixtures are prepared at a total lipid concentration of 1 mM in 200 proof ethanol (Fisher Bioreagents, Pennsylvania, USA) using a molar ratio of 50:38.5:10:1.5, ionizable lipid (DLin-MC3-DMA or ALC-0315), cholesterol, phospholipid (DSPC or DOPE), and DMG-PEG_2000_, respectively. RNA solution is added at an ionizable lipid to RNA ratio (w/w) of 60:1. Before mixing, lipid solution is warmed at 60 °C for one minute, then briefly sonicated, to ensure homogenous solubilization of lipid components. Fluids are dispensed using 1 mL sterile plastic syringes (Air-Tite, Virginia, USA), with flow controlled by syringes pumps (Harvard Apparatus, Massachusetts, USA) at a total flow rate of 4 mL/min (3:1, aqueous to organic). For example, 150 µL RNA aqueous mixture is dispensed at a flow rate of 3 mL/min, and 50 µL of lipid organic mixture is dispensed at 1 mL/min, for a final sample volume of 200 µL. Upon exiting the microfluidic outlet, the sample is immediately diluted in 1 mL phosphate buffered saline (0.01 M, 20% sucrose, pH 7.4) to bring the ethanol concentration below 5% (v/v)^34^. Each lipid mixture and RNA LNP combination is synthesized three times to assess variability. Samples are stored at 4 °C.

Before characterization steps, nanoparticles are washed and concentrated using Amicon Ultra-4 centrifugal filter units (100 kDa, UFC8100) from Millipore Sigma (Massachusetts, USA). Centrifugal filter units are first rinsed with 2 mL PBS via centrifugation at 1500 rcf for 15 min. Filtrate is discarded, and LNP sample is added to top compartment of filter unit and centrifuged for 10 min at 1000 rcf. Filtrate is discarded and 1 mL PBS is added to sample compartment then centrifuged again for 10–15 min at 1000 rcf. Samples are resuspended in PBS to a final volume of 250 µL.

### Lipid nanoparticle characterization

Size analysis is done via dynamic light scattering (DLS) analysis (Zetasizer Nano ZS90, Malvern Panalytical, Malvern, UK). For DLS sample preparation, 100 µL of washed sample is added to 1 mL PBS and filtered using 1 mL sterile plastic syringes and 0.2 µm PVDF Titan syringe filters (42204-PV, Thermo Fisher Scientific). Three measurements are obtained per sample, from which average and standard deviation values are calculated.

Encapsulation efficiency is determined via modified Quant-it™ RiboGreen Assay (Thermo Fisher Scientific) using black, opaque, 96-microwell plates (CulturPlate-96 F, Perkin Elmer, Massachusetts, USA). RiboGreen reagent binds RNA, producing a fluorescence signal proportional to the concentration, which is quantified using a standard curve. Standard curves are generated, in duplicate, by twofold serial dilution of RNA working solution corresponding to the LNP cargo (total of 6 points per standard curve). Samples are added to ensure the theoretical concentration falls near the mid-point of the standard curve. Dilutions are performed using 1 × TE buffer (10 mM Tris–HCl, 1 mM EDTA). For each sample, the concentration of unencapsulated RNA (U_*[RNA]*_) is measured by adding RiboGreen reagent to wells containing intact LNPs. The total amount of RNA (*T*_*[RNA]*_) in each sample is measured by adding RiboGreen reagent to wells containing LNPs that have been disrupted using 0.5% Triton X-100 in 1 × TE buffer (Thermo Scientific). Fluorescence measurements are collected using a Tecan Spark plate reader (Männedorf, Switzerland) with ʎ_ex_ = 485 nm (15 nm bandpass) and ʎ_em_ = 528 nm (20 nm bandpass). Encapsulated RNA (*E*_*[RNA]*_) is calculated by subtracting the unencapsulated concentration of RNA from the total concentration of RNA (Eq. 1). Encapsulation efficiency (EE%) is calculated by taking the ratio of encapsulated RNA to total RNA (Eq. 2). Each sample is tested twice. Average and standard deviation values for each lipid mixture and RNA LNP combination are calculated from measurements of three replicates. Encapsulation efficiency based on input RNA (EE_input_%%) is calculated based on LNP synthesis input RNA concentration rather than the determined total output RNA concentration. Rather than taking the ratio of encapsulated RNA to total RNA (both measured after synthesis and processing steps), EE_input_% is calculated according to Eq. (3), by taking the ratio of encapsulated RNA to input RNA concentration (*I*_*[RNA]*_*)*.

1 $${E}_{\left[RNA\right]}= {T}_{[RNA]}-{U}_{\left[RNA\right]},$$

2 $$EE\%=\frac{{E}_{[RNA]}}{{T}_{[RNA]}}\times 100,$$

3 $${EE}_{input}\%= \frac{{E}_{[RNA]}}{{I}_{[RNA]}}\times 100.$$

### Statistical analysis

Statistical analysis is performed using two measurements from three repeats of each set of reaction conditions (N = 6) for EE% and EE_input_%. One-way ANOVA and Tukey test is performed to compare EE% and EE_input_% values for MC3 vs ALC, and DSPC vs. DOPE. Analysis performed using OriginPro®2023b.

### Supplementary Information

Supplementary Information.

## Data Availability

All data generated or analysed during this study are included in this published article [and its supplementary information files].
