# Supplementary material for: A careful look at lipid nanoparticle characterization: analysis of benchmark formulations for encapsulation of RNA cargo size gradient
Source: Sci Rep. 2024 Jan 29;14:2403. doi: 10.1038/s41598-024-52685-1 (PMC10824725; doi:10.1038/s41598-024-52685-1)
Supplement: Supplementary file 1 — Supplementary Information. [file 41598_2024_52685_MOESM1_ESM.pdf]

## Supporting Information

A Careful Look at Lipid Nanoparticle Characterization: Analysis of Benchmark Formulations for Encapsulation of RNA Cargo Size Gradient

Gretchen B. Schober[1], Sandra Story[1], Dev P. Arya[1,2*]

[1]NUBAD LLC, Greer, 29650, USA
[2]Department of Chemistry, Clemson University, Clemson, 29631, USA
*Correspondence and requests for materials should be addressed to D.P.A. (email: dparya@clemson.edu)

**Supplementary Information Includes:**

Tables S1-S8: One-way ANOVA statistical analysis of encapsulation efficiency (EE%) and encapsulation efficiency based on RNA input concentration ($EE_{input}$%) for all lipid mix and RNA cargo combinations.

Tables S9-S16 Tukey test statistical analysis of encapsulation efficiency (EE%) and encapsulation efficiency based on RNA input concentration ($EE_{input}$%) for all lipid mix and RNA cargo combinations.

Figure S1: Encapsulation efficiency bar plots of DLin-MC3-DMA vs. ALC0315, and DSPC vs. DOPE.

Figure S2: Comparison of EE% and $EE_{input}$% for 0.5-10 mM total lipid concentrations

**Table S1** One way ANOVA comparing EE% values for Dlin-MC3-DMA lipid mix combinations.

| | DF | Sum of Squares | Mean Square | F Value | Prob>F |
|---|---|---|---|---|---|
| Model | 9 | 0.08885 | 0.00987 | 45.21141 | <0.0001 |
| Error | 50 | 0.01092 | 2.18E-04 | | |
| Total | 59 | 0.09976 | | | |

**Null Hypothesis: The means of all levels are equal.**

**Alternative Hypothesis: The means of one or more levels are different.**

**At the 0.05 level, the population means are significantly different.**

**Table S2** Overall ANOVA comparing $EE_{input}$% values for Dlin-MC3-DMA lipid mix combinations.

| | DF | Sum of Squares | Mean Square | F Value | Prob>F |
|---|---|---|---|---|---|
| Model | 9 | 1.01678 | 0.11298 | 90.24874 | <0.0001 |
| Error | 50 | 0.06259 | 0.00125 | | |
| Total | 59 | 1.07937 | | | |

**Null Hypothesis: The means of all levels are equal.**

**Alternative Hypothesis: The means of one or more levels are different.**

**At the 0.05 level, the population means are significantly different.**

**Table S3** Overall ANOVA comparing EE% values for ALC-0315 lipid mix combinations.

| | DF | Sum of Squares | Mean Square | F Value | Prob>F |
|---|---|---|---|---|---|
| Model | 9 | 0.04152 | 0.00461 | 18.86175 | <0.0001 |
| Error | 50 | 0.01223 | 2.45E-04 | | |
| Total | 59 | 0.05374 | | | |

**Null Hypothesis: The means of all levels are equal.**

**Alternative Hypothesis: The means of one or more levels are different.**

**At the 0.05 level, the population means are significantly different.**

**Table S4** Overall ANOVA comparing $EE_{input}$% values for ALC-0315 lipid mix combinations.

| | DF | Sum of Squares | Mean Square | F Value | Prob>F |
|---|---|---|---|---|---|
| Model | 9 | 0.51329 | 0.05703 | 31.7634 | <0.0001 |
| Error | 50 | 0.08978 | 0.0018 | | |
| Total | 59 | 0.60307 | | | |

**Null Hypothesis: The means of all levels are equal.**

**Alternative Hypothesis: The means of one or more levels are different.**

**At the 0.05 level, the population means are significantly different.**

**Table S5** Overall ANOVA comparing EE% values for DSPC lipid mix combinations

| | DF | Sum of Squares | Mean Square | F Value | Prob>F |
|---|---|---|---|---|---|
| Model | 9 | 0.07855 | 0.00873 | 31.14065 | <0.0001 |
| Error | 50 | 0.01401 | 2.80E-04 | | |
| Total | 59 | 0.09256 | | | |

**Null Hypothesis: The means of all levels are equal.**

**Alternative Hypothesis: The means of one or more levels are different.**

**At the 0.05 level, the population means are significantly different.**

**Table S6** Overall ANOVA comparing $EE_{input}$% values for DSPC lipid mix combinations.

| | DF | Sum of Squares | Mean Square | F Value | Prob>F |
|---|---|---|---|---|---|
| Model | 9 | 0.90921 | 0.10102 | 56.75899 | <0.0001 |
| Error | 50 | 0.08899 | 0.00178 | | |
| Total | 59 | 0.99821 | | | |

**Null Hypothesis: The means of all levels are equal.**

**Alternative Hypothesis: The means of one or more levels are different.**

**At the 0.05 level, the population means are significantly different.**

**Table S7** Overall ANOVA comparing EE% values for DOPE lipid mix combinations

| | DF | Sum of Squares | Mean Square | F Value | Prob>F |
|---|---|---|---|---|---|
| Model | 9 | 0.05355 | 0.00595 | 32.57278 | <0.0001 |
| Error | 50 | 0.00913 | 1.83E-04 | | |
| Total | 59 | 0.06268 | | | |

**Null Hypothesis: The means of all levels are equal.**

**Alternative Hypothesis: The means of one or more levels are different.**

**At the 0.05 level, the population means are significantly different.**

**Table S8** Overall ANOVA comparing $EE_{input}$% values for DOPE lipid mix combinations.

| | DF | Sum of Squares | Mean Square | F Value | Prob>F |
|---|---|---|---|---|---|
| Model | 9 | 0.69157 | 0.07684 | 60.62424 | <0.0001 |
| Error | 50 | 0.06337 | 0.00127 | | |
| Total | 59 | 0.75494 | | | |

**Null Hypothesis: The means of all levels are equal.**

**Alternative Hypothesis: The means of one or more levels are different.**

**At the 0.05 level, the population means are significantly different.**

**Table S9** Tukey Test comparing EE% values for Dlin-MC3-DMA lipid mix combinations. Pairs are defined by RNA type and phospholipid (DSPC or DOPE).

| | MeanDiff | SEM | q Value | Prob | Alpha | Sig | LCL | UCL |
|---|---|---|---|---|---|---|---|---|
| (10 base DOPE) (10 base DSPC) | 1.15E-02 | 8.53E-03 | 1.9 | 0.93708 | 0.05 | 0 | -1.68E-02 | 3.97E-02 |
| (21 base pair DSPC) (10 base DSPC) | 5.24E-02 | 8.53E-03 | 8.7 | <0.0001 | 0.05 | 1 | 2.41E-02 | 8.06E-02 |
| (21 base pair DSPC) (10 base DOPE) | 4.09E-02 | 8.53E-03 | 6.8 | 5.94E-04 | 0.05 | 1 | 1.26E-02 | 6.91E-02 |
| (21 base pair DOPE) (10 base DSPC) | 5.22E-02 | 8.53E-03 | 8.7 | <0.0001 | 0.05 | 1 | 2.40E-02 | 8.05E-02 |
| (21 base pair DOPE) (10 base DOPE) | 4.07E-02 | 8.53E-03 | 6.8 | 6.31E-04 | 0.05 | 1 | 1.25E-02 | 6.90E-02 |
| (21 base pair DOPE) (21 base pair DSPC) | -1.54E-04 | 8.53E-03 | 0.0 | 1 | 0.05 | 0 | -2.84E-02 | 2.81E-02 |
| (96 base DSPC) (10 base DSPC) | 1.15E-01 | 8.53E-03 | 19.0 | <0.0001 | 0.05 | 1 | 8.66E-02 | 1.43E-01 |
| (96 base DSPC) (10 base DOPE) | 1.03E-01 | 8.53E-03 | 17.1 | <0.0001 | 0.05 | 1 | 7.51E-02 | 1.32E-01 |
| (96 base DSPC) (21 base pair DSPC) | 6.24E-02 | 8.53E-03 | 10.3 | <0.0001 | 0.05 | 1 | 3.42E-02 | 9.07E-02 |
| (96 base DSPC) (21 base pair DOPE) | 6.26E-02 | 8.53E-03 | 10.4 | <0.0001 | 0.05 | 1 | 3.43E-02 | 9.08E-02 |
| (96 base DOPE) (10 base DSPC) | 1.06E-01 | 8.53E-03 | 17.5 | <0.0001 | 0.05 | 1 | 7.76E-02 | 1.34E-01 |
| (96 base DOPE) (10 base DOPE) | 9.44E-02 | 8.53E-03 | 15.6 | <0.0001 | 0.05 | 1 | 6.61E-02 | 1.23E-01 |
| (96 base DOPE) (21 base pair DSPC) | 5.35E-02 | 8.53E-03 | 8.9 | <0.0001 | 0.05 | 1 | 2.53E-02 | 8.17E-02 |
| (96 base DOPE) (21 base pair DOPE) | 5.37E-02 | 8.53E-03 | 8.9 | <0.0001 | 0.05 | 1 | 2.54E-02 | 8.19E-02 |
| (96 base DOPE) (96 base DSPC) | -8.92E-03 | 8.53E-03 | 1.5 | 0.98762 | 0.05 | 0 | -3.72E-02 | 1.93E-02 |
| (996 base DSPC) (10 base DSPC) | 1.03E-01 | 8.53E-03 | 17.1 | <0.0001 | 0.05 | 1 | 7.47E-02 | 1.31E-01 |
| (996 base DSPC) (10 base DOPE) | 9.14E-02 | 8.53E-03 | 15.2 | <0.0001 | 0.05 | 1 | 6.32E-02 | 1.20E-01 |
| (996 base DSPC) (21 base pair DSPC) | 5.05E-02 | 8.53E-03 | 8.4 | <0.0001 | 0.05 | 1 | 2.23E-02 | 7.88E-02 |
| (996 base DSPC) (21 base pair DOPE) | 5.07E-02 | 8.53E-03 | 8.4 | <0.0001 | 0.05 | 1 | 2.24E-02 | 7.89E-02 |
| (996 base DSPC) (96 base DSPC) | -1.19E-02 | 8.53E-03 | 2.0 | 0.92313 | 0.05 | 0 | -4.01E-02 | 1.63E-02 |
| (996 base DSPC) (96 base DOPE) | -2.98E-03 | 8.53E-03 | 0.5 | 1 | 0.05 | 0 | -3.12E-02 | 2.53E-02 |
| (996 base DOPE) (10 base DSPC) | 9.52E-02 | 8.53E-03 | 15.8 | <0.0001 | 0.05 | 1 | 6.69E-02 | 1.23E-01 |
| (996 base DOPE) (10 base DOPE) | 8.37E-02 | 8.53E-03 | 13.9 | <0.0001 | 0.05 | 1 | 5.54E-02 | 1.12E-01 |
| (996 base DOPE) (21 base pair DSPC) | 4.28E-02 | 8.53E-03 | 7.1 | 2.80E-04 | 0.05 | 1 | 1.46E-02 | 7.10E-02 |
| (996 base DOPE) (21 base pair DOPE) | 4.30E-02 | 8.53E-03 | 7.1 | 2.64E-04 | 0.05 | 1 | 1.47E-02 | 7.12E-02 |
| (996 base DOPE) (96 base DSPC) | -1.96E-02 | 8.53E-03 | 3.3 | 0.40768 | 0.05 | 0 | -4.79E-02 | 8.63E-03 |
| (996 base DOPE) (96 base DOPE) | -1.07E-02 | 8.53E-03 | 1.8 | 0.95924 | 0.05 | 0 | -3.89E-02 | 1.76E-02 |
| (996 base DOPE) (996 base DSPC) | -7.71E-03 | 8.53E-03 | 1.3 | 0.99565 | 0.05 | 0 | -3.60E-02 | 2.05E-02 |
| (1929 base DSPC) (10 base DSPC) | 9.48E-02 | 8.53E-03 | 15.7 | <0.0001 | 0.05 | 1 | 6.66E-02 | 1.23E-01 |
| (1929 base DSPC) (10 base DOPE) | 8.33E-02 | 8.53E-03 | 13.8 | <0.0001 | 0.05 | 1 | 5.51E-02 | 1.12E-01 |
| (1929 base DSPC) (21 base pair DSPC) | 4.24E-02 | 8.53E-03 | 7.0 | 3.26E-04 | 0.05 | 1 | 1.42E-02 | 7.07E-02 |
| (1929 base DSPC) (21 base pair DOPE) | 4.26E-02 | 8.53E-03 | 7.1 | 3.07E-04 | 0.05 | 1 | 1.43E-02 | 7.08E-02 |
| (1929 base DSPC) (96 base DSPC) | -2.00E-02 | 8.53E-03 | 3.3 | 0.38052 | 0.05 | 0 | -4.82E-02 | 8.25E-03 |
| (1929 base DSPC) (96 base DOPE) | -1.11E-02 | 8.53E-03 | 1.8 | 0.94948 | 0.05 | 0 | -3.93E-02 | 1.72E-02 |
| (1929 base DSPC) (996 base DSPC) | -8.09E-03 | 8.53E-03 | 1.3 | 0.9938 | 0.05 | 0 | -3.63E-02 | 2.02E-02 |
| (1929 base DSPC) (996 base DOPE) | -3.83E-04 | 8.53E-03 | 0.1 | 1 | 0.05 | 0 | -2.86E-02 | 2.79E-02 |
| (1929 base DOPE) (10 base DSPC) | 8.61E-02 | 8.53E-03 | 14.3 | <0.0001 | 0.05 | 1 | 5.79E-02 | 1.14E-01 |
| (1929 base DOPE) (10 base DOPE) | 7.46E-02 | 8.53E-03 | 12.4 | <0.0001 | 0.05 | 1 | 4.64E-02 | 1.03E-01 |
| (1929 base DOPE) (21 base pair DSPC) | 3.37E-02 | 8.53E-03 | 5.6 | 0.0084 | 0.05 | 1 | 5.48E-03 | 6.20E-02 |
| (1929 base DOPE) (21 base pair DOPE) | 3.39E-02 | 8.53E-03 | 5.6 | 0.00797 | 0.05 | 1 | 5.64E-03 | 6.21E-02 |
| (1929 base DOPE) (96 base DSPC) | -2.87E-02 | 8.53E-03 | 4.8 | 0.04365 | 0.05 | 1 | -5.69E-02 | -4.50E-04 |
| (1929 base DOPE) (96 base DOPE) | -1.98E-02 | 8.53E-03 | 3.3 | 0.39639 | 0.05 | 0 | -4.80E-02 | 8.47E-03 |
| (1929 base DOPE) (996 base DSPC) | -1.68E-02 | 8.53E-03 | 2.8 | 0.62422 | 0.05 | 0 | -4.50E-02 | 1.15E-02 |
| (1929 base DOPE) (996 base DOPE) | -9.08E-03 | 8.53E-03 | 1.5 | 0.98603 | 0.05 | 0 | -3.73E-02 | 1.92E-02 |
| (1929 base DOPE) (1929 base DSPC) | -8.70E-03 | 8.53E-03 | 1.4 | 0.98965 | 0.05 | 0 | -3.69E-02 | 1.96E-02 |

**Table S10** Tukey Test comparing $EE_{input}$% values for Dlin-MC3-DMA lipid mix combinations. Pairs are defined by RNA type and phospholipid (DSPC or DOPE).

| | MeanDiff | SEM | q Value | Prob | Alpha | Sig | LCL | UCL |
|---|---|---|---|---|---|---|---|---|
| (10 base DOPE) (10 base DSPC) | 2.64E-02 | 2.04E-02 | 1.8 | 0.95139 | 0.05 | 0 | -4.13E-02 | 9.40E-02 |
| (21 base pair DSPC) (10 base DSPC) | 9.62E-02 | 2.04E-02 | 6.7 | 7.86E-04 | 0.05 | 1 | 2.85E-02 | 1.64E-01 |
| (21 base pair DSPC) (10 base DOPE) | 6.98E-02 | 2.04E-02 | 4.8 | 0.03786 | 0.05 | 1 | 2.19E-03 | 1.37E-01 |
| (21 base pair DOPE) (10 base DSPC) | 8.81E-02 | 2.04E-02 | 6.1 | 0.00278 | 0.05 | 1 | 2.05E-02 | 1.56E-01 |
| (21 base pair DOPE) (10 base DOPE) | 6.18E-02 | 2.04E-02 | 4.3 | 0.10038 | 0.05 | 0 | -5.85E-03 | 1.29E-01 |
| (21 base pair DOPE) (21 base pair DSPC) | -8.03E-03 | 2.04E-02 | 0.6 | 0.99999 | 0.05 | 0 | -7.57E-02 | 5.96E-02 |
| (96 base DSPC) (10 base DSPC) | -7.87E-02 | 2.04E-02 | 5.4 | 0.01127 | 0.05 | 1 | -1.46E-01 | -1.11E-02 |
| (96 base DSPC) (10 base DOPE) | -1.05E-01 | 2.04E-02 | 7.3 | 1.83E-04 | 0.05 | 1 | -1.73E-01 | -3.74E-02 |
| (96 base DSPC) (21 base pair DSPC) | -1.75E-01 | 2.04E-02 | 12.1 | <0.0001 | 0.05 | 1 | -2.42E-01 | -1.07E-01 |
| (96 base DSPC) (21 base pair DOPE) | -1.67E-01 | 2.04E-02 | 11.6 | <0.0001 | 0.05 | 1 | -2.34E-01 | -9.92E-02 |
| (96 base DOPE) (10 base DSPC) | 2.57E-02 | 2.04E-02 | 1.8 | 0.95837 | 0.05 | 0 | -4.19E-02 | 9.33E-02 |
| (96 base DOPE) (10 base DOPE) | -6.60E-04 | 2.04E-02 | 0.0 | 1 | 0.05 | 0 | -6.83E-02 | 6.70E-02 |
| (96 base DOPE) (21 base pair DSPC) | -7.05E-02 | 2.04E-02 | 4.9 | 0.03475 | 0.05 | 1 | -1.38E-01 | -2.85E-03 |
| (96 base DOPE) (21 base pair DOPE) | -6.24E-02 | 2.04E-02 | 4.3 | 0.09311 | 0.05 | 0 | -1.30E-01 | 5.18E-03 |
| (96 base DOPE) (96 base DSPC) | 1.04E-01 | 2.04E-02 | 7.2 | 2.04E-04 | 0.05 | 1 | 3.68E-02 | 1.72E-01 |
| (996 base DSPC) (10 base DSPC) | 1.85E-01 | 2.04E-02 | 12.8 | <0.0001 | 0.05 | 1 | 1.17E-01 | 2.52E-01 |
| (996 base DSPC) (10 base DOPE) | 1.58E-01 | 2.04E-02 | 11.0 | <0.0001 | 0.05 | 1 | 9.08E-02 | 2.26E-01 |
| (996 base DSPC) (21 base pair DSPC) | 8.86E-02 | 2.04E-02 | 6.1 | 0.00258 | 0.05 | 1 | 2.10E-02 | 1.56E-01 |
| (996 base DSPC) (21 base pair DOPE) | 9.67E-02 | 2.04E-02 | 6.7 | 7.26E-04 | 0.05 | 1 | 2.90E-02 | 1.64E-01 |
| (996 base DSPC) (96 base DSPC) | 2.63E-01 | 2.04E-02 | 18.2 | <0.0001 | 0.05 | 1 | 1.96E-01 | 3.31E-01 |
| (996 base DSPC) (96 base DOPE) | 1.59E-01 | 2.04E-02 | 11.0 | <0.0001 | 0.05 | 1 | 9.15E-02 | 2.27E-01 |
| (996 base DOPE) (10 base DSPC) | 3.18E-01 | 2.04E-02 | 22.0 | <0.0001 | 0.05 | 1 | 2.51E-01 | 3.86E-01 |
| (996 base DOPE) (10 base DOPE) | 2.92E-01 | 2.04E-02 | 20.2 | <0.0001 | 0.05 | 1 | 2.24E-01 | 3.59E-01 |
| (996 base DOPE) (21 base pair DSPC) | 2.22E-01 | 2.04E-02 | 15.4 | <0.0001 | 0.05 | 1 | 1.54E-01 | 2.90E-01 |
| (996 base DOPE) (21 base pair DOPE) | 2.30E-01 | 2.04E-02 | 15.9 | <0.0001 | 0.05 | 1 | 1.62E-01 | 2.98E-01 |
| (996 base DOPE) (96 base DSPC) | 3.97E-01 | 2.04E-02 | 27.5 | <0.0001 | 0.05 | 1 | 3.29E-01 | 4.65E-01 |
| (996 base DOPE) (96 base DOPE) | 2.93E-01 | 2.04E-02 | 20.3 | <0.0001 | 0.05 | 1 | 2.25E-01 | 3.60E-01 |
| (996 base DOPE) (996 base DSPC) | 1.33E-01 | 2.04E-02 | 9.2 | <0.0001 | 0.05 | 1 | 6.58E-02 | 2.01E-01 |
| (1929 base DSPC) (10 base DSPC) | 2.78E-01 | 2.04E-02 | 19.2 | <0.0001 | 0.05 | 1 | 2.10E-01 | 3.45E-01 |
| (1929 base DSPC) (10 base DOPE) | 2.51E-01 | 2.04E-02 | 17.4 | <0.0001 | 0.05 | 1 | 1.84E-01 | 3.19E-01 |
| (1929 base DSPC) (21 base pair DSPC) | 1.82E-01 | 2.04E-02 | 12.6 | <0.0001 | 0.05 | 1 | 1.14E-01 | 2.49E-01 |
| (1929 base DSPC) (21 base pair DOPE) | 1.90E-01 | 2.04E-02 | 13.1 | <0.0001 | 0.05 | 1 | 1.22E-01 | 2.57E-01 |
| (1929 base DSPC) (96 base DSPC) | 3.56E-01 | 2.04E-02 | 24.7 | <0.0001 | 0.05 | 1 | 2.89E-01 | 4.24E-01 |
| (1929 base DSPC) (96 base DOPE) | 2.52E-01 | 2.04E-02 | 17.4 | <0.0001 | 0.05 | 1 | 1.84E-01 | 3.20E-01 |
| (1929 base DSPC) (996 base DSPC) | 9.29E-02 | 2.04E-02 | 6.4 | 0.00132 | 0.05 | 1 | 2.53E-02 | 1.61E-01 |
| (1929 base DSPC) (996 base DOPE) | -4.05E-02 | 2.04E-02 | 2.8 | 0.61328 | 0.05 | 0 | -1.08E-01 | 2.71E-02 |
| (1929 base DOPE) (10 base DSPC) | 2.87E-01 | 2.04E-02 | 19.9 | <0.0001 | 0.05 | 1 | 2.20E-01 | 3.55E-01 |
| (1929 base DOPE) (10 base DOPE) | 2.61E-01 | 2.04E-02 | 18.1 | <0.0001 | 0.05 | 1 | 1.94E-01 | 3.29E-01 |
| (1929 base DOPE) (21 base pair DSPC) | 1.91E-01 | 2.04E-02 | 13.2 | <0.0001 | 0.05 | 1 | 1.24E-01 | 2.59E-01 |
| (1929 base DOPE) (21 base pair DOPE) | 1.99E-01 | 2.04E-02 | 13.8 | <0.0001 | 0.05 | 1 | 1.32E-01 | 2.67E-01 |
| (1929 base DOPE) (96 base DSPC) | 3.66E-01 | 2.04E-02 | 25.4 | <0.0001 | 0.05 | 1 | 2.99E-01 | 4.34E-01 |
| (1929 base DOPE) (96 base DOPE) | 2.62E-01 | 2.04E-02 | 18.1 | <0.0001 | 0.05 | 1 | 1.94E-01 | 3.29E-01 |
| (1929 base DOPE) (996 base DSPC) | 1.03E-01 | 2.04E-02 | 7.1 | 2.71E-04 | 0.05 | 1 | 3.51E-02 | 1.70E-01 |
| (1929 base DOPE) (996 base DOPE) | -3.08E-02 | 2.04E-02 | 2.1 | 0.88374 | 0.05 | 0 | -9.84E-02 | 3.69E-02 |
| (1929 base DOPE) (1929 base DSPC) | 9.79E-03 | 2.04E-02 | 0.7 | 0.99997 | 0.05 | 0 | -5.78E-02 | 7.74E-02 |

**Table S**11 Tukey Test and overall one-way ANOVA comparing EE% values for ALC-0315 lipid mix combinations. Pairs are defined by RNA type and phospholipid (DSPC or DOPE).

| | MeanDiff | SEM | q Value | Prob | Alpha | Sig | LCL | UCL |
|---|---|---|---|---|---|---|---|---|
| (10 base DOPE) (10 base DSPC) | 1.68E-02 | 9.03E-03 | 2.6 | 0.6965 | 0.05 | 0 | -1.31E-02 | 4.67E-02 |
| (21 base pair DSPC) (10 base DSPC) | 2.91E-02 | 9.03E-03 | 4.6 | 0.06255 | 0.05 | 0 | -8.03E-04 | 5.90E-02 |
| (21 base pair DSPC) (10 base DOPE) | 1.23E-02 | 9.03E-03 | 1.9 | 0.93229 | 0.05 | 0 | -1.76E-02 | 4.22E-02 |
| (21 base pair DOPE) (10 base DSPC) | 2.58E-02 | 9.03E-03 | 4.0 | 0.14521 | 0.05 | 0 | -4.08E-03 | 5.57E-02 |
| (21 base pair DOPE) (10 base DOPE) | 9.04E-03 | 9.03E-03 | 1.4 | 0.99084 | 0.05 | 0 | -2.08E-02 | 3.89E-02 |
| (21 base pair DOPE) (21 base pair DSPC) | -3.27E-03 | 9.03E-03 | 0.5 | 1 | 0.05 | 0 | -3.32E-02 | 2.66E-02 |
| (96 base DSPC) (10 base DSPC) | 6.94E-02 | 9.03E-03 | 10.9 | <0.0001 | 0.05 | 1 | 3.95E-02 | 9.93E-02 |
| (96 base DSPC) (10 base DOPE) | 5.26E-02 | 9.03E-03 | 8.2 | <0.0001 | 0.05 | 1 | 2.27E-02 | 8.25E-02 |
| (96 base DSPC) (21 base pair DSPC) | 4.03E-02 | 9.03E-03 | 6.3 | 0.00173 | 0.05 | 1 | 1.04E-02 | 7.02E-02 |
| (96 base DSPC) (21 base pair DOPE) | 4.36E-02 | 9.03E-03 | 6.8 | 5.32E-04 | 0.05 | 1 | 1.37E-02 | 7.35E-02 |
| (96 base DOPE) (10 base DSPC) | 5.74E-02 | 9.03E-03 | 9.0 | <0.0001 | 0.05 | 1 | 2.75E-02 | 8.73E-02 |
| (96 base DOPE) (10 base DOPE) | 4.06E-02 | 9.03E-03 | 6.4 | 0.00156 | 0.05 | 1 | 1.07E-02 | 7.05E-02 |
| (96 base DOPE) (21 base pair DSPC) | 2.83E-02 | 9.03E-03 | 4.4 | 0.07769 | 0.05 | 0 | -1.60E-03 | 5.82E-02 |
| (96 base DOPE) (21 base pair DOPE) | 3.16E-02 | 9.03E-03 | 4.9 | 0.03078 | 0.05 | 1 | 1.67E-03 | 6.15E-02 |
| (96 base DOPE) (96 base DSPC) | -1.20E-02 | 9.03E-03 | 1.9 | 0.94147 | 0.05 | 0 | -4.19E-02 | 1.79E-02 |
| (996 base DSPC) (10 base DSPC) | 5.55E-02 | 9.03E-03 | 8.7 | <0.0001 | 0.05 | 1 | 2.56E-02 | 8.54E-02 |
| (996 base DSPC) (10 base DOPE) | 3.87E-02 | 9.03E-03 | 6.1 | 0.00304 | 0.05 | 1 | 8.81E-03 | 6.86E-02 |
| (996 base DSPC) (21 base pair DSPC) | 2.64E-02 | 9.03E-03 | 4.1 | 0.12661 | 0.05 | 0 | -3.51E-03 | 5.63E-02 |
| (996 base DSPC) (21 base pair DOPE) | 2.97E-02 | 9.03E-03 | 4.6 | 0.05344 | 0.05 | 0 | -2.37E-04 | 5.95E-02 |
| (996 base DSPC) (96 base DSPC) | -1.39E-02 | 9.03E-03 | 2.2 | 0.86845 | 0.05 | 0 | -4.38E-02 | 1.60E-02 |
| (996 base DSPC) (96 base DOPE) | -1.91E-03 | 9.03E-03 | 0.3 | 1 | 0.05 | 0 | -3.18E-02 | 2.80E-02 |
| (996 base DOPE) (10 base DSPC) | 6.04E-02 | 9.03E-03 | 9.5 | <0.0001 | 0.05 | 1 | 3.05E-02 | 9.03E-02 |
| (996 base DOPE) (10 base DOPE) | 4.37E-02 | 9.03E-03 | 6.8 | 5.16E-04 | 0.05 | 1 | 1.38E-02 | 7.35E-02 |
| (996 base DOPE) (21 base pair DSPC) | 3.13E-02 | 9.03E-03 | 4.9 | 0.03287 | 0.05 | 1 | 1.45E-03 | 6.12E-02 |
| (996 base DOPE) (21 base pair DOPE) | 3.46E-02 | 9.03E-03 | 5.4 | 0.01194 | 0.05 | 1 | 4.72E-03 | 6.45E-02 |
| (996 base DOPE) (96 base DSPC) | -8.96E-03 | 9.03E-03 | 1.4 | 0.99142 | 0.05 | 0 | -3.89E-02 | 2.09E-02 |
| (996 base DOPE) (96 base DOPE) | 3.05E-03 | 9.03E-03 | 0.5 | 1 | 0.05 | 0 | -2.68E-02 | 3.29E-02 |
| (996 base DOPE) (996 base DSPC) | 4.96E-03 | 9.03E-03 | 0.8 | 0.99992 | 0.05 | 0 | -2.49E-02 | 3.49E-02 |
| (1929 base DSPC) (10 base DSPC) | 7.93E-02 | 9.03E-03 | 12.4 | <0.0001 | 0.05 | 1 | 4.94E-02 | 1.09E-01 |
| (1929 base DSPC) (10 base DOPE) | 6.26E-02 | 9.03E-03 | 9.8 | <0.0001 | 0.05 | 1 | 3.27E-02 | 9.25E-02 |
| (1929 base DSPC) (21 base pair DSPC) | 5.02E-02 | 9.03E-03 | 7.9 | <0.0001 | 0.05 | 1 | 2.04E-02 | 8.01E-02 |
| (1929 base DSPC) (21 base pair DOPE) | 5.35E-02 | 9.03E-03 | 8.4 | <0.0001 | 0.05 | 1 | 2.36E-02 | 8.34E-02 |
| (1929 base DSPC) (96 base DSPC) | 9.94E-03 | 9.03E-03 | 1.6 | 0.98234 | 0.05 | 0 | -1.99E-02 | 3.98E-02 |
| (1929 base DSPC) (96 base DOPE) | 2.20E-02 | 9.03E-03 | 3.4 | 0.32984 | 0.05 | 0 | -7.93E-03 | 5.19E-02 |
| (1929 base DSPC) (996 base DSPC) | 2.39E-02 | 9.03E-03 | 3.7 | 0.22548 | 0.05 | 0 | -6.02E-03 | 5.38E-02 |
| (1929 base DSPC) (996 base DOPE) | 1.89E-02 | 9.03E-03 | 3.0 | 0.54015 | 0.05 | 0 | -1.10E-02 | 4.88E-02 |
| (1929 base DOPE) (10 base DSPC) | 8.05E-02 | 9.03E-03 | 12.6 | <0.0001 | 0.05 | 1 | 5.06E-02 | 1.10E-01 |
| (1929 base DOPE) (10 base DOPE) | 6.37E-02 | 9.03E-03 | 10.0 | <0.0001 | 0.05 | 1 | 3.39E-02 | 9.36E-02 |
| (1929 base DOPE) (21 base pair DSPC) | 5.14E-02 | 9.03E-03 | 8.1 | <0.0001 | 0.05 | 1 | 2.15E-02 | 8.13E-02 |
| (1929 base DOPE) (21 base pair DOPE) | 5.47E-02 | 9.03E-03 | 8.6 | <0.0001 | 0.05 | 1 | 2.48E-02 | 8.46E-02 |
| (1929 base DOPE) (96 base DSPC) | 1.11E-02 | 9.03E-03 | 1.7 | 0.96337 | 0.05 | 0 | -1.88E-02 | 4.10E-02 |
| (1929 base DOPE) (96 base DOPE) | 2.31E-02 | 9.03E-03 | 3.6 | 0.2623 | 0.05 | 0 | -6.75E-03 | 5.30E-02 |
| (1929 base DOPE) (996 base DSPC) | 2.50E-02 | 9.03E-03 | 3.9 | 0.17376 | 0.05 | 0 | -4.84E-03 | 5.49E-02 |
| (1929 base DOPE) (996 base DOPE) | 2.01E-02 | 9.03E-03 | 3.1 | 0.4544 | 0.05 | 0 | -9.80E-03 | 5.00E-02 |
| (1929 base DOPE) (1929 base DSPC) | 1.18E-03 | 9.03E-03 | 0.2 | 1 | 0.05 | 0 | -2.87E-02 | 3.11E-02 |

**Table S12** Tukey Test comparing $EE_{input}$% values for ALC-0315 lipid mix combinations. Pairs are defined by RNA type and phospholipid (DSPC or DOPE).

| | MeanDiff | SEM | q Value | Prob | Alpha | Sig | LCL | UCL |
|---|---|---|---|---|---|---|---|---|
| (10 base DOPE) (10 base DSPC) | -4.32E-02 | 2.45E-02 | 2.5 | 0.75348 | 0.05 | 0 | -1.24E-01 | 3.78E-02 |
| (21 base pair DSPC) (10 base DSPC) | 9.40E-02 | 2.45E-02 | 5.4 | 0.0117 | 0.05 | 1 | 1.30E-02 | 1.75E-01 |
| (21 base pair DSPC) (10 base DOPE) | 1.37E-01 | 2.45E-02 | 7.9 | <0.0001 | 0.05 | 1 | 5.61E-02 | 2.18E-01 |
| (21 base pair DOPE) (10 base DSPC) | 4.42E-02 | 2.45E-02 | 2.6 | 0.72924 | 0.05 | 0 | -3.68E-02 | 1.25E-01 |
| (21 base pair DOPE) (10 base DOPE) | 8.73E-02 | 2.45E-02 | 5.0 | 0.02521 | 0.05 | 1 | 6.32E-03 | 1.68E-01 |
| (21 base pair DOPE) (21 base pair DSPC) | -4.98E-02 | 2.45E-02 | 2.9 | 0.57913 | 0.05 | 0 | -1.31E-01 | 3.12E-02 |
| (96 base DSPC) (10 base DSPC) | 7.81E-02 | 2.45E-02 | 4.5 | 0.06723 | 0.05 | 0 | -2.89E-03 | 1.59E-01 |
| (96 base DSPC) (10 base DOPE) | 1.21E-01 | 2.45E-02 | 7.0 | 3.44E-04 | 0.05 | 1 | 4.03E-02 | 2.02E-01 |
| (96 base DSPC) (21 base pair DSPC) | -1.59E-02 | 2.45E-02 | 0.9 | 0.99968 | 0.05 | 0 | -9.68E-02 | 6.51E-02 |
| (96 base DSPC) (21 base pair DOPE) | 3.40E-02 | 2.45E-02 | 2.0 | 9.25E-01 | 0.05 | 0 | -4.70E-02 | 1.15E-01 |
| (96 base DOPE) (10 base DSPC) | 3.66E-02 | 2.45E-02 | 2.1 | 0.88796 | 0.05 | 0 | -4.44E-02 | 1.18E-01 |
| (96 base DOPE) (10 base DOPE) | 7.97E-02 | 2.45E-02 | 4.6 | 0.05696 | 0.05 | 0 | -1.26E-03 | 1.61E-01 |
| (96 base DOPE) (21 base pair DSPC) | -5.74E-02 | 2.45E-02 | 3.3 | 0.37943 | 0.05 | 0 | -1.38E-01 | 2.36E-02 |
| (96 base DOPE) (21 base pair DOPE) | -7.58E-03 | 2.45E-02 | 0.4 | 1 | 0.05 | 0 | -8.86E-02 | 7.34E-02 |
| (96 base DOPE) (96 base DSPC) | -4.15E-02 | 2.45E-02 | 2.4 | 0.79151 | 0.05 | 0 | -1.23E-01 | 3.95E-02 |
| (996 base DSPC) (10 base DSPC) | 2.58E-01 | 2.45E-02 | 14.9 | <0.0001 | 0.05 | 1 | 1.77E-01 | 3.38E-01 |
| (996 base DSPC) (10 base DOPE) | 3.01E-01 | 2.45E-02 | 17.4 | <0.0001 | 0.05 | 1 | 2.20E-01 | 3.82E-01 |
| (996 base DSPC) (21 base pair DSPC) | 1.64E-01 | 2.45E-02 | 9.5 | <0.0001 | 0.05 | 1 | 8.26E-02 | 2.45E-01 |
| (996 base DSPC) (21 base pair DOPE) | 2.13E-01 | 2.45E-02 | 12.3 | <0.0001 | 0.05 | 1 | 1.32E-01 | 2.94E-01 |
| (996 base DSPC) (96 base DSPC) | 1.79E-01 | 2.45E-02 | 10.4 | <0.0001 | 0.05 | 1 | 9.84E-02 | 2.60E-01 |
| (996 base DSPC) (96 base DOPE) | 2.21E-01 | 2.45E-02 | 12.8 | <0.0001 | 0.05 | 1 | 1.40E-01 | 3.02E-01 |
| (996 base DOPE) (10 base DSPC) | 1.99E-01 | 2.45E-02 | 11.5 | <0.0001 | 0.05 | 1 | 1.18E-01 | 2.80E-01 |
| (996 base DOPE) (10 base DOPE) | 2.42E-01 | 2.45E-02 | 14.0 | <0.0001 | 0.05 | 1 | 1.61E-01 | 3.23E-01 |
| (996 base DOPE) (21 base pair DSPC) | 1.05E-01 | 2.45E-02 | 6.1 | 0.00307 | 0.05 | 1 | 2.38E-02 | 1.86E-01 |
| (996 base DOPE) (21 base pair DOPE) | 1.55E-01 | 2.45E-02 | 8.9 | <0.0001 | 0.05 | 1 | 7.36E-02 | 2.36E-01 |
| (996 base DOPE) (96 base DSPC) | 1.21E-01 | 2.45E-02 | 7.0 | 3.74E-04 | 0.05 | 1 | 3.97E-02 | 2.02E-01 |
| (996 base DOPE) (96 base DOPE) | 1.62E-01 | 2.45E-02 | 9.4 | <0.0001 | 0.05 | 1 | 8.12E-02 | 2.43E-01 |
| (996 base DOPE) (996 base DSPC) | -5.88E-02 | 2.45E-02 | 3.4 | 0.34652 | 0.05 | 0 | -1.40E-01 | 2.22E-02 |
| (1929 base DSPC) (10 base DSPC) | 2.12E-01 | 2.45E-02 | 12.3 | <0.0001 | 0.05 | 1 | 1.31E-01 | 2.93E-01 |
| (1929 base DSPC) (10 base DOPE) | 2.55E-01 | 2.45E-02 | 14.8 | <0.0001 | 0.05 | 1 | 1.74E-01 | 3.36E-01 |
| (1929 base DSPC) (21 base pair DSPC) | 1.18E-01 | 2.45E-02 | 6.8 | 5.32E-04 | 0.05 | 1 | 3.71E-02 | 1.99E-01 |
| (1929 base DSPC) (21 base pair DOPE) | 1.68E-01 | 2.45E-02 | 9.7 | <0.0001 | 0.05 | 1 | 8.69E-02 | 2.49E-01 |
| (1929 base DSPC) (96 base DSPC) | 1.34E-01 | 2.45E-02 | 7.7 | <0.0001 | 0.05 | 1 | 5.29E-02 | 2.15E-01 |
| (1929 base DSPC) (96 base DOPE) | 1.75E-01 | 2.45E-02 | 10.1 | <0.0001 | 0.05 | 1 | 9.45E-02 | 2.56E-01 |
| (1929 base DSPC) (996 base DSPC) | -4.55E-02 | 2.45E-02 | 2.6 | 0.69469 | 0.05 | 0 | -1.26E-01 | 3.55E-02 |
| (1929 base DSPC) (996 base DOPE) | 1.33E-02 | 2.45E-02 | 0.8 | 0.99993 | 0.05 | 0 | -6.77E-02 | 9.43E-02 |
| (1929 base DOPE) (10 base DSPC) | 1.17E-01 | 2.45E-02 | 6.8 | 6.05E-04 | 0.05 | 1 | 3.61E-02 | 1.98E-01 |
| (1929 base DOPE) (10 base DOPE) | 1.60E-01 | 2.45E-02 | 9.3 | <0.0001 | 0.05 | 1 | 7.93E-02 | 2.41E-01 |
| (1929 base DOPE) (21 base pair DSPC) | 2.32E-02 | 2.45E-02 | 1.3 | 0.99391 | 0.05 | 0 | -5.78E-02 | 1.04E-01 |
| (1929 base DOPE) (21 base pair DOPE) | 7.30E-02 | 2.45E-02 | 4.2 | 0.11051 | 0.05 | 0 | -8.03E-03 | 1.54E-01 |
| (1929 base DOPE) (96 base DSPC) | 3.90E-02 | 2.45E-02 | 2.3 | 0.84446 | 0.05 | 0 | -4.20E-02 | 1.20E-01 |
| (1929 base DOPE) (96 base DOPE) | 8.05E-02 | 2.45E-02 | 4.7 | 0.05237 | 0.05 | 0 | -4.46E-04 | 1.62E-01 |
| (1929 base DOPE) (996 base DSPC) | -1.40E-01 | 2.45E-02 | 8.1 | <0.0001 | 0.05 | 1 | -2.21E-01 | -5.94E-02 |
| (1929 base DOPE) (996 base DOPE) | -8.16E-02 | 2.45E-02 | 4.7 | 0.0467 | 0.05 | 1 | -1.63E-01 | -6.51E-04 |
| (1929 base DOPE) (1929 base DSPC) | -9.49E-02 | 2.45E-02 | 5.5 | 0.01044 | 0.05 | 1 | -1.76E-01 | -1.39E-02 |

**Table S13** Tukey Test comparing EE% for DSPC lipid mix combinations. Pairs are defined by RNA type and ionizable lipid (ALC or MC3).

| | MeanDiff | SEM | q Value | Prob | Alpha | Sig | LCL | UCL |
|---|---|---|---|---|---|---|---|---|
| (10 base ALC) (10 base MC3) | 3.18E-02 | 9.67E-03 | 4.6 | 0.05292 | 0.05 | 0 | -2.16E-04 | 6.38E-02 |
| (21 base pair MC3) (10 base MC3) | 5.24E-02 | 9.67E-03 | 7.7 | <0.0001 | 0.05 | 1 | 2.04E-02 | 8.44E-02 |
| (21 base pair MC3) (10 base ALC) | 2.06E-02 | 9.67E-03 | 3.0 | 0.5154 | 0.05 | 0 | -1.14E-02 | 5.26E-02 |
| (21 base pair ALC) (10 base MC3) | 6.09E-02 | 9.67E-03 | 8.9 | <0.0001 | 0.05 | 1 | 2.89E-02 | 9.29E-02 |
| (21 base pair ALC) (10 base ALC) | 2.91E-02 | 9.67E-03 | 4.3 | 0.10389 | 0.05 | 0 | -2.91E-03 | 6.11E-02 |
| (21 base pair ALC) (21 base pair MC3) | 8.49E-03 | 9.67E-03 | 1.2 | 0.9965 | 0.05 | 0 | -2.35E-02 | 4.05E-02 |
| (96 base MC3) (10 base MC3) | 1.15E-01 | 9.67E-03 | 16.8 | <0.0001 | 0.05 | 1 | 8.28E-02 | 1.47E-01 |
| (96 base MC3) (10 base ALC) | 8.30E-02 | 9.67E-03 | 12.1 | <0.0001 | 0.05 | 1 | 5.10E-02 | 1.15E-01 |
| (96 base MC3) (21 base pair MC3) | 6.24E-02 | 9.67E-03 | 9.1 | <0.0001 | 0.05 | 1 | 3.04E-02 | 9.44E-02 |
| (96 base MC3) (21 base pair ALC) | 5.39E-02 | 9.67E-03 | 7.9 | <0.0001 | 0.05 | 1 | 2.19E-02 | 8.59E-02 |
| (96 base ALC) (10 base MC3) | 1.01E-01 | 9.67E-03 | 14.8 | <0.0001 | 0.05 | 1 | 6.92E-02 | 1.33E-01 |
| (96 base ALC) (10 base ALC) | 6.94E-02 | 9.67E-03 | 10.2 | <0.0001 | 0.05 | 1 | 3.74E-02 | 1.01E-01 |
| (96 base ALC) (21 base pair MC3) | 4.88E-02 | 9.67E-03 | 7.1 | 2.53E-04 | 0.05 | 1 | 1.68E-02 | 8.08E-02 |
| (96 base ALC) (21 base pair ALC) | 4.03E-02 | 9.67E-03 | 5.9 | 0.00437 | 0.05 | 1 | 8.30E-03 | 7.23E-02 |
| (96 base ALC) (96 base MC3) | -1.36E-02 | 9.67E-03 | 2.0 | 0.91832 | 0.05 | 0 | -4.56E-02 | 1.84E-02 |
| (996 base MC3) (10 base MC3) | 1.03E-01 | 9.67E-03 | 15.1 | <0.0001 | 0.05 | 1 | 7.09E-02 | 1.35E-01 |
| (996 base MC3) (10 base ALC) | 7.11E-02 | 9.67E-03 | 10.4 | <0.0001 | 0.05 | 1 | 3.91E-02 | 1.03E-01 |
| (996 base MC3) (21 base pair MC3) | 5.05E-02 | 9.67E-03 | 7.4 | 1.38E-04 | 0.05 | 1 | 1.85E-02 | 8.25E-02 |
| (996 base MC3) (21 base pair ALC) | 4.20E-02 | 9.67E-03 | 6.1 | 0.0025 | 0.05 | 1 | 1.00E-02 | 7.40E-02 |
| (996 base MC3) (96 base MC3) | -1.19E-02 | 9.67E-03 | 1.7 | 0.9635 | 0.05 | 0 | -4.39E-02 | 2.01E-02 |
| (996 base MC3) (96 base ALC) | 1.73E-03 | 9.67E-03 | 0.3 | 1 | 0.05 | 0 | -3.03E-02 | 3.37E-02 |
| (996 base ALC) (10 base MC3) | 8.72E-02 | 9.67E-03 | 12.8 | <0.0001 | 0.05 | 1 | 5.53E-02 | 1.19E-01 |
| (996 base ALC) (10 base ALC) | 5.55E-02 | 9.67E-03 | 8.1 | <0.0001 | 0.05 | 1 | 2.35E-02 | 8.75E-02 |
| (996 base ALC) (21 base pair MC3) | 3.49E-02 | 9.67E-03 | 5.1 | 0.02267 | 0.05 | 1 | 2.87E-03 | 6.69E-02 |
| (996 base ALC) (21 base pair ALC) | 2.64E-02 | 9.67E-03 | 3.9 | 0.19028 | 0.05 | 0 | -5.62E-03 | 5.84E-02 |
| (996 base ALC) (96 base MC3) | -2.76E-02 | 9.67E-03 | 4.0 | 0.14788 | 0.05 | 0 | -5.95E-02 | 4.45E-03 |
| (996 base ALC) (96 base ALC) | -1.39E-02 | 9.67E-03 | 2.0 | 0.90818 | 0.05 | 0 | -4.59E-02 | 1.81E-02 |
| (996 base ALC) (996 base MC3) | -1.57E-02 | 9.67E-03 | 2.3 | 0.83256 | 0.05 | 0 | -4.76E-02 | 1.64E-02 |
| (1929 base MC3) (10 base MC3) | 9.48E-02 | 9.67E-03 | 13.9 | <0.0001 | 0.05 | 1 | 6.28E-02 | 1.27E-01 |
| (1929 base MC3) (10 base ALC) | 6.30E-02 | 9.67E-03 | 9.2 | <0.0001 | 0.05 | 1 | 3.10E-02 | 9.50E-02 |
| (1929 base MC3) (21 base pair MC3) | 4.24E-02 | 9.67E-03 | 6.2 | 0.0022 | 0.05 | 1 | 1.04E-02 | 7.44E-02 |
| (1929 base MC3) (21 base pair ALC) | 3.39E-02 | 9.67E-03 | 5.0 | 0.02952 | 0.05 | 1 | 1.94E-03 | 6.59E-02 |
| (1929 base MC3) (96 base MC3) | -2.00E-02 | 9.67E-03 | 2.9 | 0.55699 | 0.05 | 0 | -5.20E-02 | 1.20E-02 |
| (1929 base MC3) (96 base ALC) | -6.37E-03 | 9.67E-03 | 0.9 | 1.00E+00 | 0.05 | 0 | -3.84E-02 | 2.56E-02 |
| (1929 base MC3) (996 base MC3) | -8.09E-03 | 9.67E-03 | 1.2 | 0.99755 | 0.05 | 0 | -4.01E-02 | 2.39E-02 |
| (1929 base MC3) (996 base ALC) | 7.55E-03 | 9.67E-03 | 1.1 | 0.99856 | 0.05 | 0 | -2.44E-02 | 3.96E-02 |
| (1929 base ALC) (10 base MC3) | 1.11E-01 | 9.67E-03 | 16.3 | <0.0001 | 0.05 | 1 | 7.91E-02 | 1.43E-01 |
| (1929 base ALC) (10 base ALC) | 7.93E-02 | 9.67E-03 | 11.6 | <0.0001 | 0.05 | 1 | 4.73E-02 | 1.11E-01 |
| (1929 base ALC) (21 base pair MC3) | 5.87E-02 | 9.67E-03 | 8.6 | <0.0001 | 0.05 | 1 | 2.67E-02 | 9.07E-02 |
| (1929 base ALC) (21 base pair ALC) | 5.02E-02 | 9.67E-03 | 7.4 | 1.52E-04 | 0.05 | 1 | 1.83E-02 | 8.22E-02 |
| (1929 base ALC) (96 base MC3) | -3.68E-03 | 9.67E-03 | 0.5 | 1 | 0.05 | 0 | -3.57E-02 | 2.83E-02 |
| (1929 base ALC) (96 base ALC) | 9.94E-03 | 9.67E-03 | 1.5 | 0.98894 | 0.05 | 0 | -2.21E-02 | 4.19E-02 |
| (1929 base ALC) (996 base MC3) | 8.22E-03 | 9.67E-03 | 1.2 | 0.99726 | 0.05 | 0 | -2.38E-02 | 4.02E-02 |
| (1929 base ALC) (996 base ALC) | 2.39E-02 | 9.67E-03 | 3.5 | 0.3097 | 0.05 | 0 | -8.13E-03 | 5.59E-02 |
| (1929 base ALC) (1929 base MC3) | 1.63E-02 | 9.67E-03 | 2.4 | 0.79698 | 0.05 | 0 | -1.57E-02 | 4.83E-02 |

**Table S14** Tukey Test comparing $EE_{input}$% values for DSPC lipid mix combinations. Pairs are defined by RNA type and ionizable lipid (ALC or MC3).

| | MeanDiff | SEM | q Value | Prob | Alpha | Sig | LCL | UCL |
|---|---|---|---|---|---|---|---|---|
| (10 base ALC) (10 base MC3) | 7.17E-02 | 2.44E-02 | 4.2 | 0.12077 | 0.05 | 0 | -8.95E-03 | 1.52E-01 |
| (21 base pair MC3) (10 base MC3) | 9.62E-02 | 2.44E-02 | 5.6 | 0.00853 | 0.05 | 1 | 1.55E-02 | 1.77E-01 |
| (21 base pair MC3) (10 base ALC) | 2.45E-02 | 2.44E-02 | 1.4 | 0.99063 | 0.05 | 0 | -5.62E-02 | 1.05E-01 |
| (21 base pair ALC) (10 base MC3) | 1.66E-01 | 2.44E-02 | 9.6 | <0.0001 | 0.05 | 1 | 8.50E-02 | 2.46E-01 |
| (21 base pair ALC) (10 base ALC) | 9.40E-02 | 2.44E-02 | 5.5 | 0.01114 | 0.05 | 1 | 1.33E-02 | 1.75E-01 |
| (21 base pair ALC) (21 base pair MC3) | 6.95E-02 | 2.44E-02 | 4.0 | 0.14732 | 0.05 | 0 | -1.12E-02 | 1.50E-01 |
| (96 base MC3) (10 base MC3) | -7.87E-02 | 2.44E-02 | 4.6 | 0.061 | 0.05 | 0 | -1.59E-01 | 1.92E-03 |
| (96 base MC3) (10 base ALC) | -1.50E-01 | 2.44E-02 | 8.7 | <0.0001 | 0.05 | 1 | -2.31E-01 | -6.98E-02 |
| (96 base MC3) (21 base pair MC3) | -1.75E-01 | 2.44E-02 | 10.2 | <0.0001 | 0.05 | 1 | -2.56E-01 | -9.42E-02 |
| (96 base MC3) (21 base pair ALC) | -2.44E-01 | 2.44E-02 | 14.2 | <0.0001 | 0.05 | 1 | -3.25E-01 | -1.64E-01 |
| (96 base ALC) (10 base MC3) | 1.50E-01 | 2.44E-02 | 8.7 | <0.0001 | 0.05 | 1 | 6.91E-02 | 2.30E-01 |
| (96 base ALC) (10 base ALC) | 7.81E-02 | 2.44E-02 | 4.5 | 0.06494 | 0.05 | 0 | -2.54E-03 | 1.59E-01 |
| (96 base ALC) (21 base pair MC3) | 5.36E-02 | 2.44E-02 | 3.1 | 0.46947 | 0.05 | 0 | -2.70E-02 | 1.34E-01 |
| (96 base ALC) (21 base pair ALC) | -1.59E-02 | 2.44E-02 | 0.9 | 0.99967 | 0.05 | 0 | -9.65E-02 | 6.48E-02 |
| (96 base ALC) (96 base MC3) | 2.28E-01 | 2.44E-02 | 13.3 | <0.0001 | 0.05 | 1 | 1.48E-01 | 3.09E-01 |
| (996 base MC3) (10 base MC3) | 1.85E-01 | 2.44E-02 | 10.7 | <0.0001 | 0.05 | 1 | 1.04E-01 | 2.65E-01 |
| (996 base MC3) (10 base ALC) | 1.13E-01 | 2.44E-02 | 6.6 | 9.70E-04 | 0.05 | 1 | 3.25E-02 | 1.94E-01 |
| (996 base MC3) (21 base pair MC3) | 8.86E-02 | 2.44E-02 | 5.1 | 0.0208 | 0.05 | 1 | 7.99E-03 | 1.69E-01 |
| (996 base MC3) (21 base pair ALC) | 1.92E-02 | 2.44E-02 | 1.1 | 0.9985 | 0.05 | 0 | -6.15E-02 | 9.98E-02 |
| (996 base MC3) (96 base MC3) | 2.63E-01 | 2.44E-02 | 15.3 | <0.0001 | 0.05 | 1 | 1.83E-01 | 3.44E-01 |
| (996 base MC3) (96 base ALC) | 3.50E-02 | 2.44E-02 | 2.0 | 0.90927 | 0.05 | 0 | -4.56E-02 | 1.16E-01 |
| (996 base ALC) (10 base MC3) | 3.29E-01 | 2.44E-02 | 19.1 | <0.0001 | 0.05 | 1 | 2.49E-01 | 4.10E-01 |
| (996 base ALC) (10 base ALC) | 2.58E-01 | 2.44E-02 | 15.0 | <0.0001 | 0.05 | 1 | 1.77E-01 | 3.38E-01 |
| (996 base ALC) (21 base pair MC3) | 2.33E-01 | 2.44E-02 | 13.5 | <0.0001 | 0.05 | 1 | 1.52E-01 | 3.14E-01 |
| (996 base ALC) (21 base pair ALC) | 1.64E-01 | 2.44E-02 | 9.5 | <0.0001 | 0.05 | 1 | 8.29E-02 | 2.44E-01 |
| (996 base ALC) (96 base MC3) | 4.08E-01 | 2.44E-02 | 23.7 | <0.0001 | 0.05 | 1 | 3.27E-01 | 4.89E-01 |
| (996 base ALC) (96 base ALC) | 1.79E-01 | 2.44E-02 | 10.4 | <0.0001 | 0.05 | 1 | 9.88E-02 | 2.60E-01 |
| (996 base ALC) (996 base MC3) | 1.44E-01 | 2.44E-02 | 8.4 | <0.0001 | 0.05 | 1 | 6.38E-02 | 2.25E-01 |
| (1929 base MC3) (10 base MC3) | 2.78E-01 | 2.44E-02 | 16.1 | <0.0001 | 0.05 | 1 | 1.97E-01 | 3.58E-01 |
| (1929 base MC3) (10 base ALC) | 2.06E-01 | 2.44E-02 | 12.0 | <0.0001 | 0.05 | 1 | 1.25E-01 | 2.87E-01 |
| (1929 base MC3) (21 base pair MC3) | 1.82E-01 | 2.44E-02 | 10.5 | <0.0001 | 0.05 | 1 | 1.01E-01 | 2.62E-01 |
| (1929 base MC3) (21 base pair ALC) | 1.12E-01 | 2.44E-02 | 6.5 | 0.00112 | 0.05 | 1 | 3.14E-02 | 1.93E-01 |
| (1929 base MC3) (96 base MC3) | 3.56E-01 | 2.44E-02 | 20.7 | <0.0001 | 0.05 | 1 | 2.76E-01 | 4.37E-01 |
| (1929 base MC3) (96 base ALC) | 1.28E-01 | 2.44E-02 | 7.4 | 1.26E-04 | 0.05 | 1 | 4.73E-02 | 2.09E-01 |
| (1929 base MC3) (996 base MC3) | 9.29E-02 | 2.44E-02 | 5.4 | 0.01263 | 0.05 | 1 | 1.23E-02 | 1.74E-01 |
| (1929 base MC3) (996 base ALC) | -5.15E-02 | 2.44E-02 | 3.0 | 0.5263 | 0.05 | 0 | -1.32E-01 | 2.91E-02 |
| (1929 base ALC) (10 base MC3) | 2.84E-01 | 2.44E-02 | 16.5 | <0.0001 | 0.05 | 1 | 2.03E-01 | 3.64E-01 |
| (1929 base ALC) (10 base ALC) | 2.12E-01 | 2.44E-02 | 12.3 | <0.0001 | 0.05 | 1 | 1.31E-01 | 2.93E-01 |
| (1929 base ALC) (21 base pair MC3) | 1.88E-01 | 2.44E-02 | 10.9 | <0.0001 | 0.05 | 1 | 1.07E-01 | 2.68E-01 |
| (1929 base ALC) (21 base pair ALC) | 1.18E-01 | 2.44E-02 | 6.9 | 4.96E-04 | 0.05 | 1 | 3.74E-02 | 1.99E-01 |
| (1929 base ALC) (96 base MC3) | 3.62E-01 | 2.44E-02 | 21.0 | <0.0001 | 0.05 | 1 | 2.82E-01 | 4.43E-01 |
| (1929 base ALC) (96 base ALC) | 1.34E-01 | 2.44E-02 | 7.8 | <0.0001 | 0.05 | 1 | 5.33E-02 | 2.15E-01 |
| (1929 base ALC) (996 base MC3) | 9.89E-02 | 2.44E-02 | 5.7 | 0.00608 | 0.05 | 1 | 1.83E-02 | 1.80E-01 |
| (1929 base ALC) (996 base ALC) | -4.55E-02 | 2.44E-02 | 2.6 | 0.68949 | 0.05 | 0 | -1.26E-01 | 3.51E-02 |
| (1929 base ALC) (1929 base MC3) | 6.01E-03 | 2.44E-02 | 0.3 | 1 | 0.05 | 0 | -7.46E-02 | 8.66E-02 |

**Table S15** Tukey Test comparing EE% values for DOPE lipid mix combinations. Pairs are defined by RNA type and ionizable lipid (ALC or MC3).

| | MeanDiff | SEM | q Value | Prob | Alpha | Sig | LCL | UCL |
|---|---|---|---|---|---|---|---|---|
| (10 base ALC) (10 base MC3) | 3.71E-02 | 7.80E-03 | 6.7 | 6.86E-04 | 0.05 | 1 | 1.12E-02 | 6.29E-02 |
| (21 base pair MC3) (10 base MC3) | 4.07E-02 | 7.80E-03 | 7.4 | 1.41E-04 | 0.05 | 1 | 1.49E-02 | 6.66E-02 |
| (21 base pair MC3) (10 base ALC) | 3.68E-03 | 7.80E-03 | 0.7 | 0.99998 | 0.05 | 0 | -2.22E-02 | 2.95E-02 |
| (21 base pair ALC) (10 base MC3) | 4.61E-02 | 7.80E-03 | 8.4 | <0.0001 | 0.05 | 1 | 2.03E-02 | 7.19E-02 |
| (21 base pair ALC) (10 base ALC) | 9.04E-03 | 7.80E-03 | 1.6 | 0.9752 | 0.05 | 0 | -1.68E-02 | 3.49E-02 |
| (21 base pair ALC) (21 base pair MC3) | 5.37E-03 | 7.80E-03 | 1.0 | 0.99948 | 0.05 | 0 | -2.05E-02 | 3.12E-02 |
| (96 base MC3) (10 base MC3) | 9.44E-02 | 7.80E-03 | 17.1 | <0.0001 | 0.05 | 1 | 6.86E-02 | 1.20E-01 |
| (96 base MC3) (10 base ALC) | 5.73E-02 | 7.80E-03 | 10.4 | <0.0001 | 0.05 | 1 | 3.15E-02 | 8.32E-02 |
| (96 base MC3) (21 base pair MC3) | 5.37E-02 | 7.80E-03 | 9.7 | <0.0001 | 0.05 | 1 | 2.78E-02 | 7.95E-02 |
| (96 base MC3) (21 base pair ALC) | 4.83E-02 | 7.80E-03 | 8.8 | <0.0001 | 0.05 | 1 | 2.25E-02 | 7.41E-02 |
| (96 base ALC) (10 base MC3) | 7.77E-02 | 7.80E-03 | 14.1 | <0.0001 | 0.05 | 1 | 5.18E-02 | 1.03E-01 |
| (96 base ALC) (10 base ALC) | 4.06E-02 | 7.80E-03 | 7.4 | 1.49E-04 | 0.05 | 1 | 1.48E-02 | 6.64E-02 |
| (96 base ALC) (21 base pair MC3) | 3.69E-02 | 7.80E-03 | 6.7 | 7.24E-04 | 0.05 | 1 | 1.11E-02 | 6.28E-02 |
| (96 base ALC) (21 base pair ALC) | 3.16E-02 | 7.80E-03 | 5.7 | 0.00639 | 0.05 | 1 | 5.73E-03 | 5.74E-02 |
| (96 base ALC) (96 base MC3) | -1.67E-02 | 7.80E-03 | 3.0 | 0.50757 | 0.05 | 0 | -4.26E-02 | 9.11E-03 |
| (996 base MC3) (10 base MC3) | 8.37E-02 | 7.80E-03 | 15.2 | <0.0001 | 0.05 | 1 | 5.79E-02 | 1.10E-01 |
| (996 base MC3) (10 base ALC) | 4.66E-02 | 7.80E-03 | 8.5 | <0.0001 | 0.05 | 1 | 2.08E-02 | 7.25E-02 |
| (996 base MC3) (21 base pair MC3) | 4.30E-02 | 7.80E-03 | 7.8 | <0.0001 | 0.05 | 1 | 1.71E-02 | 6.88E-02 |
| (996 base MC3) (21 base pair ALC) | 3.76E-02 | 7.80E-03 | 6.8 | 5.47E-04 | 0.05 | 1 | 1.18E-02 | 6.34E-02 |
| (996 base MC3) (96 base MC3) | -1.07E-02 | 7.80E-03 | 1.9 | 0.93057 | 0.05 | 0 | -3.65E-02 | 1.51E-02 |
| (996 base MC3) (96 base ALC) | 6.03E-03 | 7.80E-03 | 1.1 | 0.99868 | 0.05 | 0 | -1.98E-02 | 3.19E-02 |
| (996 base ALC) (10 base MC3) | 8.07E-02 | 7.80E-03 | 14.6 | <0.0001 | 0.05 | 1 | 5.49E-02 | 1.07E-01 |
| (996 base ALC) (10 base ALC) | 4.37E-02 | 7.80E-03 | 7.9 | <0.0001 | 0.05 | 1 | 1.78E-02 | 6.95E-02 |
| (996 base ALC) (21 base pair MC3) | 4.00E-02 | 7.80E-03 | 7.2 | 1.96E-04 | 0.05 | 1 | 1.42E-02 | 6.58E-02 |
| (996 base ALC) (21 base pair ALC) | 3.46E-02 | 7.80E-03 | 6.3 | 0.0019 | 0.05 | 1 | 8.78E-03 | 6.04E-02 |
| (996 base ALC) (96 base MC3) | -1.37E-02 | 7.80E-03 | 2.5 | 0.76067 | 0.05 | 0 | -3.95E-02 | 1.22E-02 |
| (996 base ALC) (96 base ALC) | 3.05E-03 | 7.80E-03 | 0.6 | 0.99999 | 0.05 | 0 | -2.28E-02 | 2.89E-02 |
| (996 base ALC) (996 base MC3) | -2.98E-03 | 7.80E-03 | 0.5 | 1 | 0.05 | 0 | -2.88E-02 | 2.29E-02 |
| (1929 base MC3) (10 base MC3) | 7.46E-02 | 7.80E-03 | 13.5 | <0.0001 | 0.05 | 1 | 4.88E-02 | 1.00E-01 |
| (1929 base MC3) (10 base ALC) | 3.76E-02 | 7.80E-03 | 6.8 | 5.55E-04 | 0.05 | 1 | 1.17E-02 | 6.34E-02 |
| (1929 base MC3) (21 base pair MC3) | 3.39E-02 | 7.80E-03 | 6.1 | 0.00255 | 0.05 | 1 | 8.05E-03 | 5.97E-02 |
| (1929 base MC3) (21 base pair ALC) | 2.85E-02 | 7.80E-03 | 5.2 | 0.01993 | 0.05 | 1 | 2.68E-03 | 5.43E-02 |
| (1929 base MC3) (96 base MC3) | -1.98E-02 | 7.80E-03 | 3.6 | 0.2764 | 0.05 | 0 | -4.56E-02 | 6.06E-03 |
| (1929 base MC3) (96 base ALC) | -3.05E-03 | 7.80E-03 | 0.6 | 1.00E+00 | 0.05 | 0 | -2.89E-02 | 2.28E-02 |
| (1929 base MC3) (996 base MC3) | -9.08E-03 | 7.80E-03 | 1.6 | 0.97459 | 0.05 | 0 | -3.49E-02 | 1.68E-02 |
| (1929 base MC3) (996 base ALC) | -6.10E-03 | 7.80E-03 | 1.1 | 0.99856 | 0.05 | 0 | -3.19E-02 | 1.97E-02 |
| (1929 base ALC) (10 base MC3) | 1.01E-01 | 7.80E-03 | 18.3 | <0.0001 | 0.05 | 1 | 7.50E-02 | 1.27E-01 |
| (1929 base ALC) (10 base ALC) | 6.37E-02 | 7.80E-03 | 11.6 | <0.0001 | 0.05 | 1 | 3.79E-02 | 8.96E-02 |
| (1929 base ALC) (21 base pair MC3) | 6.01E-02 | 7.80E-03 | 10.9 | <0.0001 | 0.05 | 1 | 3.42E-02 | 8.59E-02 |
| (1929 base ALC) (21 base pair ALC) | 5.47E-02 | 7.80E-03 | 9.9 | <0.0001 | 0.05 | 1 | 2.89E-02 | 8.05E-02 |
| (1929 base ALC) (96 base MC3) | 6.42E-03 | 7.80E-03 | 1.2 | 0.99788 | 0.05 | 0 | -1.94E-02 | 3.23E-02 |
| (1929 base ALC) (96 base ALC) | 2.31E-02 | 7.80E-03 | 4.2 | 0.11485 | 0.05 | 0 | -2.69E-03 | 4.90E-02 |
| (1929 base ALC) (996 base MC3) | 1.71E-02 | 7.80E-03 | 3.1 | 0.47519 | 0.05 | 0 | -8.72E-03 | 4.29E-02 |
| (1929 base ALC) (996 base ALC) | 2.01E-02 | 7.80E-03 | 3.6 | 0.25679 | 0.05 | 0 | -5.74E-03 | 4.59E-02 |
| (1929 base ALC) (1929 base MC3) | 2.62E-02 | 7.80E-03 | 4.7 | 0.04447 | 0.05 | 1 | 3.56E-04 | 5.20E-02 |

**Table S16** Tukey Test comparing $EE_{input}$% values for DOPE lipid mix combinations. Pairs are defined by RNA type and ionizable lipid (ALC or MC3).

| | MeanDiff | SEM | q Value | Prob | Alpha | Sig | LCL | UCL |
|---|---|---|---|---|---|---|---|---|
| (10 base ALC) (10 base MC3) | 2.17E-03 | 2.06E-02 | 0.15 | 1 | 0.05 | 0 | -6.59E-02 | 7.02E-02 |
| (21 base pair MC3) (10 base MC3) | 6.18E-02 | 2.06E-02 | 4.25 | 0.10481 | 0.05 | 0 | -6.27E-03 | 1.30E-01 |
| (21 base pair MC3) (10 base ALC) | 5.96E-02 | 2.06E-02 | 4.10 | 0.13283 | 0.05 | 0 | -8.44E-03 | 1.28E-01 |
| (21 base pair ALC) (10 base MC3) | 8.95E-02 | 2.06E-02 | 6.16 | 0.00246 | 0.05 | 1 | 2.14E-02 | 1.58E-01 |
| (21 base pair ALC) (10 base ALC) | 8.73E-02 | 2.06E-02 | 6.01 | 0.00343 | 0.05 | 1 | 1.93E-02 | 1.55E-01 |
| (21 base pair ALC) (21 base pair MC3) | 2.77E-02 | 2.06E-02 | 1.91 | 0.93691 | 0.05 | 0 | -4.03E-02 | 9.57E-02 |
| (96 base MC3) (10 base MC3) | -6.60E-04 | 2.06E-02 | 0.05 | 1 | 0.05 | 0 | -6.87E-02 | 6.74E-02 |
| (96 base MC3) (10 base ALC) | -2.83E-03 | 2.06E-02 | 0.19 | 1 | 0.05 | 0 | -7.09E-02 | 6.52E-02 |
| (96 base MC3) (21 base pair MC3) | -6.24E-02 | 2.06E-02 | 4.30 | 0.09731 | 0.05 | 0 | -1.30E-01 | 5.61E-03 |
| (96 base MC3) (21 base pair ALC) | -9.01E-02 | 2.06E-02 | 6.20 | 0.00222 | 0.05 | 1 | -1.58E-01 | -2.21E-02 |
| (96 base ALC) (10 base MC3) | 8.19E-02 | 2.06E-02 | 5.63 | 0.00765 | 0.05 | 1 | 1.39E-02 | 1.50E-01 |
| (96 base ALC) (10 base ALC) | 7.97E-02 | 2.06E-02 | 5.49 | 0.01046 | 0.05 | 1 | 1.17E-02 | 1.48E-01 |
| (96 base ALC) (21 base pair MC3) | 2.01E-02 | 2.06E-02 | 1.38 | 0.99223 | 0.05 | 0 | -4.79E-02 | 8.82E-02 |
| (96 base ALC) (21 base pair ALC) | -7.58E-03 | 2.06E-02 | 0.52 | 1 | 0.05 | 0 | -7.56E-02 | 6.05E-02 |
| (96 base ALC) (96 base MC3) | 8.26E-02 | 2.06E-02 | 5.68 | 0.00695 | 0.05 | 1 | 1.45E-02 | 1.51E-01 |
| (996 base MC3) (10 base MC3) | 2.92E-01 | 2.06E-02 | 20.08 | <0.0001 | 0.05 | 1 | 2.24E-01 | 3.60E-01 |
| (996 base MC3) (10 base ALC) | 2.90E-01 | 2.06E-02 | 19.93 | <0.0001 | 0.05 | 1 | 2.22E-01 | 3.58E-01 |
| (996 base MC3) (21 base pair MC3) | 2.30E-01 | 2.06E-02 | 15.83 | <0.0001 | 0.05 | 1 | 1.62E-01 | 2.98E-01 |
| (996 base MC3) (21 base pair ALC) | 2.02E-01 | 2.06E-02 | 13.92 | <0.0001 | 0.05 | 1 | 1.34E-01 | 2.70E-01 |
| (996 base MC3) (96 base MC3) | 2.93E-01 | 2.06E-02 | 20.13 | <0.0001 | 0.05 | 1 | 2.24E-01 | 3.61E-01 |
| (996 base MC3) (96 base ALC) | 2.10E-01 | 2.06E-02 | 14.45 | <0.0001 | 0.05 | 1 | 1.42E-01 | 2.78E-01 |
| (996 base ALC) (10 base MC3) | 2.44E-01 | 2.06E-02 | 16.79 | <0.0001 | 0.05 | 1 | 1.76E-01 | 3.12E-01 |
| (996 base ALC) (10 base ALC) | 2.42E-01 | 2.06E-02 | 16.64 | <0.0001 | 0.05 | 1 | 1.74E-01 | 3.10E-01 |
| (996 base ALC) (21 base pair MC3) | 1.82E-01 | 2.06E-02 | 12.54 | <0.0001 | 0.05 | 1 | 1.14E-01 | 2.50E-01 |
| (996 base ALC) (21 base pair ALC) | 1.55E-01 | 2.06E-02 | 10.64 | <0.0001 | 0.05 | 1 | 8.66E-02 | 2.23E-01 |
| (996 base ALC) (96 base MC3) | 2.45E-01 | 2.06E-02 | 16.84 | <0.0001 | 0.05 | 1 | 1.77E-01 | 3.13E-01 |
| (996 base ALC) (96 base ALC) | 1.62E-01 | 2.06E-02 | 11.16 | <0.0001 | 0.05 | 1 | 9.41E-02 | 2.30E-01 |
| (996 base ALC) (996 base MC3) | -4.78E-02 | 2.06E-02 | 3.29 | 0.39155 | 0.05 | 0 | -1.16E-01 | 2.02E-02 |
| (1929 base MC3) (10 base MC3) | 2.61E-01 | 2.06E-02 | 17.97 | <0.0001 | 0.05 | 1 | 1.93E-01 | 3.29E-01 |
| (1929 base MC3) (10 base ALC) | 2.59E-01 | 2.06E-02 | 17.82 | <0.0001 | 0.05 | 1 | 1.91E-01 | 3.27E-01 |
| (1929 base MC3) (21 base pair MC3) | 1.99E-01 | 2.06E-02 | 13.72 | <0.0001 | 0.05 | 1 | 1.31E-01 | 2.67E-01 |
| (1929 base MC3) (21 base pair ALC) | 1.72E-01 | 2.06E-02 | 11.81 | <0.0001 | 0.05 | 1 | 1.04E-01 | 2.40E-01 |
| (1929 base MC3) (96 base MC3) | 2.62E-01 | 2.06E-02 | 18.01 | <0.0001 | 0.05 | 1 | 1.94E-01 | 3.30E-01 |
| (1929 base MC3) (96 base ALC) | 1.79E-01 | 2.06E-02 | 12.33 | <0.0001 | 0.05 | 1 | 1.11E-01 | 2.47E-01 |
| (1929 base MC3) (996 base MC3) | -3.08E-02 | 2.06E-02 | 2.12 | 0.88746 | 0.05 | 0 | -9.88E-02 | 3.73E-02 |
| (1929 base MC3) (996 base ALC) | 1.71E-02 | 2.06E-02 | 1.17 | 0.99773 | 0.05 | 0 | -5.10E-02 | 8.51E-02 |
| (1929 base ALC) (10 base MC3) | 1.62E-01 | 2.06E-02 | 11.18 | <0.0001 | 0.05 | 1 | 9.44E-02 | 2.30E-01 |
| (1929 base ALC) (10 base ALC) | 1.60E-01 | 2.06E-02 | 11.03 | <0.0001 | 0.05 | 1 | 9.22E-02 | 2.28E-01 |
| (1929 base ALC) (21 base pair MC3) | 1.01E-01 | 2.06E-02 | 6.93 | 4.19E-04 | 0.05 | 1 | 3.26E-02 | 1.69E-01 |
| (1929 base ALC) (21 base pair ALC) | 7.30E-02 | 2.06E-02 | 5.02 | 2.66E-02 | 0.05 | 1 | 4.92E-03 | 1.41E-01 |
| (1929 base ALC) (96 base MC3) | 1.63E-01 | 2.06E-02 | 11.22 | <0.0001 | 0.05 | 1 | 9.51E-02 | 2.31E-01 |
| (1929 base ALC) (96 base ALC) | 8.05E-02 | 2.06E-02 | 5.54 | 0.00931 | 0.05 | 1 | 1.25E-02 | 1.49E-01 |
| (1929 base ALC) (996 base MC3) | -1.29E-01 | 2.06E-02 | 8.91 | <0.0001 | 0.05 | 1 | -1.97E-01 | -6.14E-02 |
| (1929 base ALC) (996 base ALC) | -8.16E-02 | 2.06E-02 | 5.62 | 0.00795 | 0.05 | 1 | -1.50E-01 | -1.36E-02 |
| (1929 base ALC) (1929 base MC3) | -9.87E-02 | 2.06E-02 | 6.79 | 5.77E-04 | 0.05 | 1 | -1.67E-01 | -3.06E-02 |

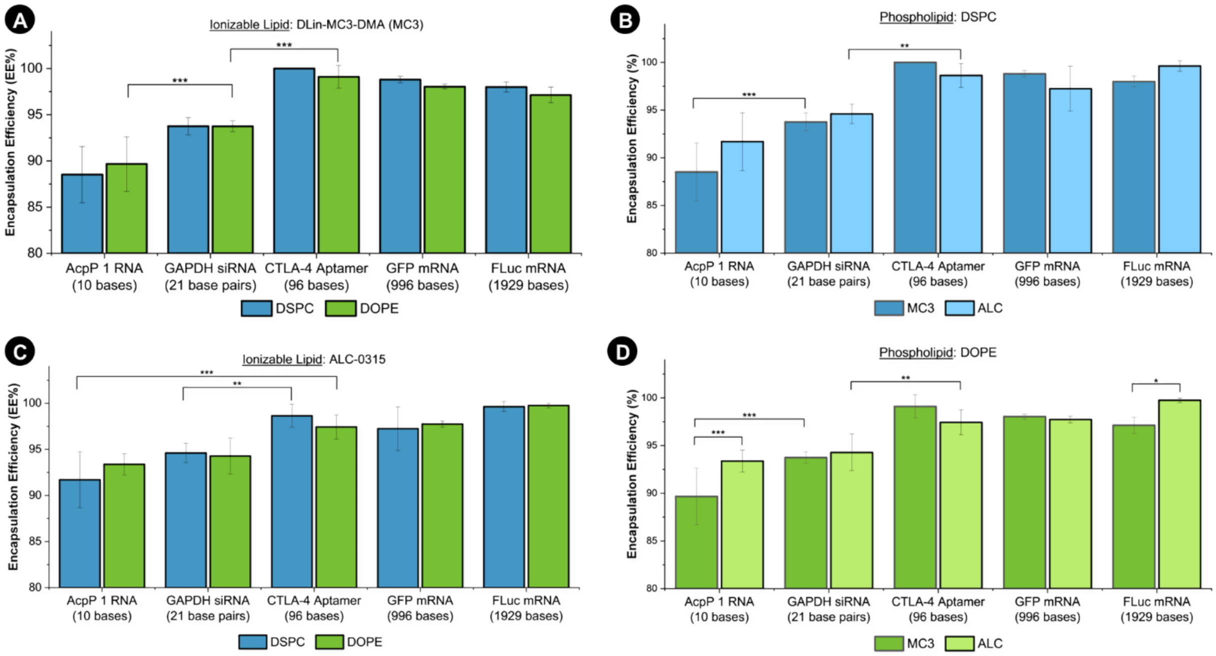


**Figure S1** Encapsulation efficiency (EE%) of lipid recipe and RNA combinations. (A) Comparison of lipid mixtures containing MC3, with blue bars representing DSPC, and green bars representing DOPE. (B) Comparison of lipid mixtures containing ALC, with blue bars representing DSPC, and green bars representing DOPE. (C) Comparison of lipid mixtures containing DSPC. (D) Comparison of lipid mixtures containing DOPE. Brackets indicate notable significant differences as determined by one-way ANOVA and Tukey test (*$P \leq 0.05$, ***$P \leq 0.001$).

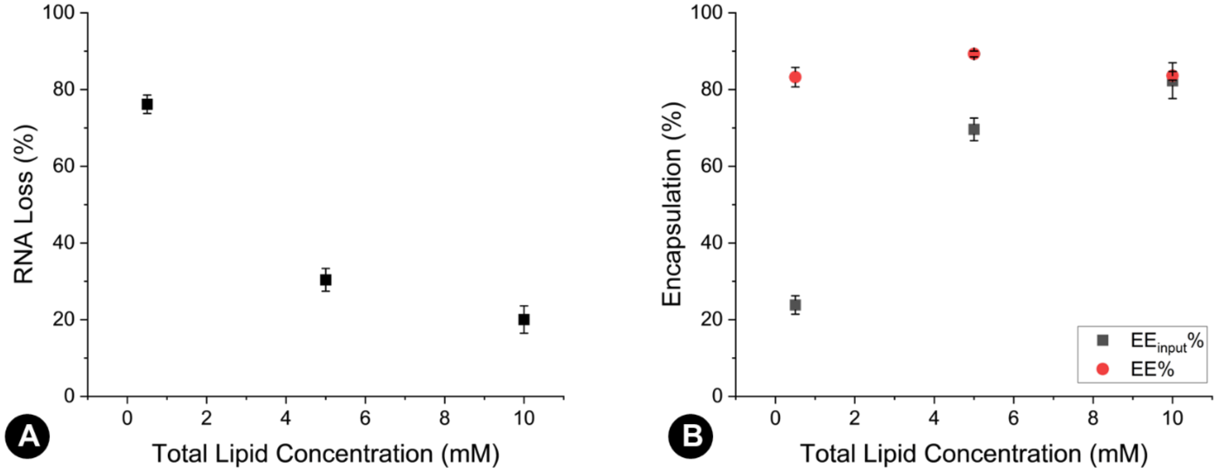


**Figure S2** Encapsulation of FLuc mRNA (1929 bases) at various total lipid concentrations, keeping ionizable lipid to RNA ratio (w/w) consistent. (**A**) As lipid concentration increases, the amount of input RNA lost decreases, as more is encapsulated. (**B**) As lipid concentration is increased, the EE% and EE$_{input}$% appear to approach convergence. Traditional calculation of EE suggests that RNA encapsulation is >70%, with negligible differences as total lipid concentration increases. When EE is calculated based on input concentration, a much more dramatic increase in encapsulation is observed with increasing lipid concentration.
